# Optimal Item Calibration for Computerized Achievement Tests

**DOI:** 10.1007/s11336-019-09673-6

**Published:** 2019-06-10

**Authors:** Mahmood Ul Hassan, Frank Miller

**Affiliations:** Stockholm University, Stockholm, Sweden

**Keywords:** achievement tests, computerized tests, item calibration, optimal restricted design, two-parameter logistic model

## Abstract

Item calibration is a technique to estimate characteristics of questions (called items) for achievement tests. In computerized tests, item calibration is an important tool for maintaining, updating and developing new items for an item bank. To efficiently sample examinees with specific ability levels for this calibration, we use optimal design theory assuming that the probability to answer correctly follows an item response model. Locally optimal unrestricted designs have usually a few design points for ability. In practice, it is hard to sample examinees from a population with these specific ability levels due to unavailability or limited availability of examinees. To counter this problem, we use the concept of optimal restricted designs and show that this concept naturally fits to item calibration. We prove an equivalence theorem needed to verify optimality of a design. Locally optimal restricted designs provide intervals of ability levels for optimal calibration of an item. When assuming a two-parameter logistic model, several scenarios with D-optimal restricted designs are presented for calibration of a single item and simultaneous calibration of several items. These scenarios show that the naive way to sample examinees around unrestricted design points is not optimal.

**Electronic supplementary material:**

The online version of this article (10.1007/s11336-019-09673-6) contains supplementary material, which is available to authorized users.

## Introduction

Achievement tests are an important part, e.g., of higher education to quantify the proficiency of examinees. An alternative of growing importance to traditional paper-and-pencil tests is computerized adaptive tests (CAT). Examinees perform the achievement tests at the computer and everyone receives a sequence of questions, called items. The advantage of CAT is that the items received can depend on the answer to previous items, e.g., examinees with many correct answers can be given more difficult questions subsequently which can then characterize their ability in more detail. By this, questions which are too hard or too easy for an examinee are avoided and “a high-quality estimate of the examinee’s proficiency can be made using as few as half as many items than in a fixed-form test” (Buyske, 2005). 

A prerequisite before administrating a CAT is the existence of a collection of items, an item bank. Based on the item bank, the CAT-algorithm can choose appropriate items for the examinees. This means that the characteristics of items, e.g., the difficulty, need to be determined before they are included into a CAT. This determination of item characteristics is called calibration of items. A common situation is that achievement tests are done periodically, e.g., year by year. Then the task is to update an item bank continuously with new items. Zheng (2014) pointed out the importance of this item replenishment and stressed the need for efficient and accurate calibration of the new items.

In principle, one could perform separate calibration studies where some voluntary test takers answer to new items. However, this is usually a quite costly option and it can be more feasible to add instead a small calibration part to an ordinary achievement test. The items from the calibration part are then available in achievement tests in future examination periods. This principle is, e.g., applied in the Swedish Scholastic Assessment Test (Universitets- och högskolerådet, 2019) which is administered as paper-and-pencil test. Adding similarly new items to be calibrated to a CAT has been called online calibration (Stocking, 1988) and Zheng (2014) reviews methods for it. Irrespectively if added to a paper-and-pencil, to a CAT, or to a non-adaptive computerized test, the calibration part has to be quite small such that the burden of this add-on part on the examinees is negligible.

We assume that an ordinary computerized test is performed (CAT or non-adaptive) and that the abilities of the examinees are well determined by their answers to a larger part of the operational items. We focus here in this work on the calibration part for new items which are seeded into the later part of operational items in a computerized test. A set of new items should be tested in the calibration and we consider here the situation that we can allocate to each examinee a small, fixed number of these new items. Our aim is to allocate these items to examinees in a good way such that we obtain high-quality estimates for the item characteristics. 

For designing the calibration part, we will apply optimal design theory, see e.g., Atkinson, Donev and Tobias (2007). The use of optimal design theory for item calibration has been discussed previously and designs have been elaborated, see e.g., Berger (1992), Buyske (2005), Lu (2014), Zheng (2014), van der Linden and Ren (2015), Ren, van der Linden and Diao (2017).

In contrast to problems in traditional optimal design setup, we have in this context not the possibility to select examinees with desired proficiency freely within a design space. This would theoretically require the access to a large number of examinees with specific abilities, a problem discussed, e.g., by Zheng (2014), van der Linden and Ren (2015) and Ren et al. (2017). The problem is avoided if sequential optimization is done. Then, for a given examinee, the best calibration item is chosen. Some achievement tests, however, test examinees in parallel and a sequential optimal design cannot be applied. In the Swedish Scholastic Assessment Test, for example, more than 60,000 examinees participate on each of two test dates per year. We consider here such a parallel testing situation, where we have at one test date a given population of examinees for the item calibration: the examinees participating in the computerized test. Based on an assumed proficiency distribution of these examinees, we will apply in this work restricted optimization to this distribution. Restricted optimization (also called constrained or bounded optimization) has been discussed in other contexts than achievement tests by Wynn (1982), Sahm and Schwabe (2000). This type of restricted optimal designs has to our knowledge not been applied for item calibration despite that it is the natural adaption of traditional optimal design to finite populations (Wynn, 1982). We are able with this method to gain general insights how item calibration can be optimized.

In the following Sect. 2, we describe the assumed model and the optimal design theory used. We will then present a new equivalence theorem which provides us with a condition to check whether a certain restricted design is optimal or not. This theorem is very general, e.g., applies to general item response models. In Sect. 2, we also describe the algorithm developed for computation of optimal designs. In Sects. 3 and 4, we compute optimal designs in several scenarios. In these scenarios, we present situations with up to three items to calibrate. In real applications, the number of items usually is much larger. We discuss in Sect. 5 an easy way to apply our results for realistic situations. We summarize our insights and conclude with a discussion (Sect. 6) where we point out directions of future research. The proof of our equivalence theorem is elaborated in an “Appendix”.

## Model for Optimal Item Calibration

### Model for Item Calibration

Item response theory (IRT) modeling has been shown to be a flexible tool for item calibration. The idea of IRT is the assumption that each examinee has an ability $\theta \in \Theta = {\mathbb {R}}$ and that the probability for an examinee with ability $\theta$ to correctly response to item *i* ($i=1,\dots ,n$) follows a non-decreasing function

1 $$\begin{aligned} p_i(\theta ) = P\left( {{{\mathrm{Y} = 1|}}\theta }, \beta _i \right) , \end{aligned}$$

where $Y = 1$ indicates a correct and $Y = 0$ an incorrect response to an item. The function $p_i$ depends on a parameter vector $\beta _i$. An assumption which often is made is $\theta \sim N(0,1)$ for the population of examinees. The basic goal of item calibration is to establish a bank of items with known item parameters $\beta _i$. The efficiency of an item calibration depends on prior knowledge of item parameters ($\beta _i$) and ability levels of examinees ($\theta$) sampled from the population who are to be allocated to the item.

#### Example 1

An important IRT model used in practice for optimal item calibration is the two-parameter logistic model. The probability for an examinee with ability $\theta$ to correctly response to item *i* ($i=1,\dots ,n$) with item parameters $\beta _i=(a_i,\,b_i)$ is defined as

2 $$\begin{aligned} p_i(\theta ) = P\left( {{{\mathrm{Y} = 1|}}\theta ,a_i,b_i} \right) = \frac{1}{{1 + {e^{ - a_i(\theta - b_i)}}}}, \end{aligned}$$

where $a_i \in \,(0,\infty )$ is called discrimination parameter, and $b_i \in {\mathbb {R}}$ is called difficulty parameter of an item. In practice, typical ranges of item parameters might be $b_i\in \,[-3,3]$, $a_i \in \,[0.3,3]$, see Buyske (2005).

### Optimal Unrestricted Design

A design for item calibration is a rule how to sample desired ability levels of examinees for estimation of unknown item parameters. We have here *n* different items to calibrate and assume that each examinee can calibrate at most one of those (see Sect. 5 for the case when each examinee calibrates $k>1$ items). First, we are interested in unrestricted designs, meaning that we have no restrictions in availability of examinees with specific ability levels; the space of examinees’ abilities is called $\Theta = {\mathbb {R}}$. Using continuous designs [see Chapter 9 in Atkinson et al. (2007)], we represent designs by probability measures $\xi$ over the design space $\chi =\Theta \times \{1,\dots ,n\}$. A $(\theta ,i) \in \chi$ means here that examinees with ability $\theta$ are sampled for item *i*. The restriction $\xi _i$ of $\xi$ to $\Theta \times \{i\}$ describes how abilities of examinees should be chosen for item *i*.

We assume to sample examinees with $m_i$ distinct ability levels $\theta _{i1},\,\theta _{i2}, \dots ,\,\theta _{im_i}$ in $\Theta$ with sample proportions (weights) $w_{i1}, \dots , w_{im_i} \ge 0$ for all items *i*, such that $\sum \nolimits _{i=1}^n\sum \nolimits _{j=1}^{m_i}w_{ij}=1$. Here, $w_{ij}$ is the sample proportion of examinees assigned to each distinct ability level $\theta _{ij}$ for item *i* and $\sum \nolimits _{j = 1}^{m_i} {w_{ij}}$ is the proportion of examinees assigned to item *i*.

In order to search for a good item calibration design $\xi$, we follow classical optimal design theory and focus on the design’s Fisher information matrix for the item parameters. This matrix indicates the precision of the model parameters estimators.

The model (1) is a generalized linear model (GLM). Its logit link $\eta _i (\theta ) = \log \left( \frac{{p_i(\theta )}}{{1 - p_i(\theta )}}\right)$ is assumed to be differentiable in $\beta _i$. Further, we assume that $\frac{{\partial \eta _i (\theta )}}{{\partial \beta _i }}$ are continuous in $\theta$. The standardized information matrix of item parameters $\beta =(\beta _1,\dots ,\beta _n)$ (Fisher’s information matrix divided by number of observations) is a block-diagonal matrix

$$\begin{aligned} M(\xi ) = \mathrm{diag}(M_1(\xi _1), \dots , M_n(\xi _n)) \hbox { with } M_i(\xi _i ) = \sum \limits _{j = 1}^{m_i} w_{ij} \nu _i (\theta _{ij})\left( \frac{{\partial \eta (\theta _{ij})}}{{\partial \beta _i }}\right) {\left( \frac{{\partial \eta (\theta _{ij})}}{{\partial \beta _i }}\right) ^T}, \end{aligned}$$

where $\nu _i (\theta ) = p_i(\theta )(1 - p_i(\theta ))$ is the weight function for this GLM [see Chapter 22 in Atkinson et al. (2007)].

In optimal design theory, we optimize some appropriate convex function $\Psi$ of $M(\xi )$. A design ${\xi ^*}$ is called $\Psi$-optimal if ${\xi ^*} = \arg \min _{\xi } \,\,\Psi \{ M(\xi )\}$. The information matrix $M(\xi )$ depends on the unknown model parameters $\beta _i, i=1, \dots ,n$. If a researcher has a best guess or initial values about the model parameters, the optimal design can be constructed based on these initial values. Such an optimal design is referred to as a locally $\,\Psi$-optimal design (Atkinson et al., 2007).

We will consider directional derivatives which tell how the information of a design $\xi$ changes in a direction of another design $\lambda$:

$$\begin{aligned} F_\Psi (\xi , \lambda ) = \mathop {\lim }\limits _{\alpha \downarrow {0}} \frac{1}{\alpha }[\,\Psi \{ M((1 - \alpha )\xi + \alpha \lambda )\} - \Psi \{ M(\xi )\} ]. \end{aligned}$$

Especially, when $\lambda$ is the measure $\delta _{(\theta ,i)}$ with unit weight at design point $\theta$ for item *i*, we can quantify how much the criterion changes when a small amount of observations in $\theta$ are added for item *i*. We write then $F_\Psi (\xi ,\theta ,i) = F_\Psi (\xi , \delta _{(\theta ,i)}).$ An optimality criterion $\Psi$ is called differentiable if all directional derivatives can be expressed as integral over directional derivatives with respect to $\delta _{(\theta ,i)}$: $F_\Psi (\xi ,\lambda ) = \int _{\chi } F_\Psi (\xi ,\theta ,i)~\lambda (d(\theta ,i))$, see e.g., Whittle (1973). We assume in this paper that criterion $\Psi$ is differentiable.

The General Equivalence Theorem (Kiefer & Wolfowitz, 1960) is an important result which gives us a way to check whether a design is $\,\Psi$-optimal among all designs. It states the equivalence of the following three conditions for the design $\xi ^*$ :

- The design ${\xi ^*}$ minimizes $\Psi \{ M(\xi )\}$.
- The minimum over $(\theta ,i) \in \chi$ of $F_\Psi ({\xi ^*},\theta , i ) \ge 0$.
- The minimum over $(\theta ,i) \in \chi$ of $F_\Psi ({\xi ^*},\theta , i ) = 0$ and it is achieved at the support-points $(\theta ,i)$ of the design ${\xi ^*}$.

Several optimality criteria $\Psi$ (e.g., E-, A-, G- and D-optimality) have been proposed in the literature (Pukelsheim, 2006). The D-optimality criterion is one of the most popular and intensively studied criteria in optimal design methodology (Berger, 1992; Silvey, 1980; Berger, King & Wong, 2000). It is also most frequently used in online items calibration literature (Chang & Lu, 2010; Jones & Jin, 1994; Zhu, 2006). Buyske (1998) has justified the use of a specific L-optimality to reduce parameter drift in online calibration. From the above-mentioned criteria, A-, D-, and L-optimalities are differentiable.

#### Example 2

We consider again the important two-parameter logistic model (2) and present link function, derivative, information matrix, and directional derivative for D-optimality, since we will illustrate our method with this model in Sects. 3 and 4. The link function is: $\eta _i (\theta ) = a_i(\theta - b_i)$ having the derivative

$$\begin{aligned} \frac{{\partial \eta _i (\theta )}}{{\partial \beta _i }} = \left[ {\begin{array}{*{20}{c}} {\frac{{\partial \eta _i (\theta )}}{{\partial a_i}}}\\ {\frac{{\partial \eta _i (\theta )}}{{\partial b_i}}} \end{array}} \right] = \left[ {\begin{array}{*{20}{c}} { (\theta - b_i)}\\ -a_i \end{array}} \right] \,\hbox {where}\,\,\,\beta _i = (a_i,b_i). \end{aligned}$$

The information matrix is:

$$\begin{aligned} M_i(\xi _i ) = \sum \limits _{j = 1}^{m_i} w_{ij} \nu _i(\theta _{ij})\left[ {\begin{array}{*{20}{c}} {{{(\theta _{ij} - b_i)}^2}}&{}\quad { - a_i(\theta _{ij} - b_i)}\\ { - a_i(\theta _{ij} - b_i)}&{}\quad {{a_i^2}} \end{array}} \right] . \end{aligned}$$

We use the D-optimality criterion in Sects. 3 and 4 which maximizes the determinant of the standardized information matrix $M(\xi )$ so that the generalized variance of the parameter estimates is minimized (Atkinson et al., 2007). Equivalently to maximizing the determinant $|M(\xi )|$ we can minimize $\Psi \{ M(\xi )\} = - \log \left| {M(\xi )} \right| = - \sum _{i=1}^n \log \left| {M_i(\xi _i )} \right|$ and the directional derivative for this criterion is then given by

$$\begin{aligned} F_D(\xi ,\theta , i ) = 2 - p_i(\theta )(1 - p_i(\theta ))\left[ {\begin{array}{*{20}{c}} {(\theta - b_i)}&{ - a_i} \end{array}} \right] M_i{(\xi _i )^{ - 1}}{\left[ {\begin{array}{*{20}{c}} {(\theta - b_i)}&{ - a_i} \end{array}} \right] ^T}. \end{aligned}$$

The directional derivative is an important part of the General Equivalence Theorem. Later in Sects. 3 and 4 we use directional derivatives for verifying the optimality of our restricted design with the Equivalence Theorem for Item Calibration which will be presented in Sect. 2.3.

### Optimal Restricted Design

Our aim is to select the best subsamples of examinees for each of *n* new items in order to optimize item calibration. Since we cannot sample a large number of examinees with specific abilities, we cannot apply directly the optimal design based on the method described in Sect. 2.2. However, we can use the main optimal design ideas described before but restrict the set of available designs using an approach initially described by Wynn (1982).

Let *g* be a continuous density on $\Theta$ which describes the examinees participating in the computerized test. A calibration design is described by sub-densities $h_0, h_1, \dots , h_n \ge 0$ on $\Theta$ where $h_1, \dots , h_n$ describe the sub-population of examinees to be assigned to Item $1, \dots , n$, respectively, and $h_0$ describes the non-sampling distribution. These sub-densities $h_i$ should describe together the whole available population *g*:

3 $$\begin{aligned}&\sum _{i=0}^n h_i(\theta ) = g(\theta ) \hbox { for all } \theta \in \Theta . \end{aligned}$$

Further, we allow to use the proportion $s \in (0,1]$ of examinees for calibration (where *s* can be 1 if there are several items to calibrate, $n \ge 2$). This restriction means for the non-sampled population $h_0$:

4 $$\begin{aligned}&\int _{\Theta } h_0(\theta )~\mathrm{d}\theta = 1-s. \end{aligned}$$

We collate the densities $h_0, h_1, \dots , h_n$ into a single density *h* on ${\tilde{\chi }} = \Theta \times \{0, 1, \dots , n\}$ defined by $h(\theta ,i) = h_i(\theta )$. The density *h* defines a probability measure $\xi$ on ${\tilde{\chi }}$ by $\xi (A)= \int _{A} h(x)~\mathrm{d}x$ for sets $A \in {\tilde{\chi }}$, but we use here in our notation *h* to represent the design.

Let $\Xi ^g_s$ be the set of all such designs *h* where the $h_i$ fulfill restrictions (3) and (4). The standardized information matrices for item *i* of a design $h_i$ ($i=1,\dots ,n$) are given by

$$\begin{aligned} M_i(h_i ) = \int \limits _{\Theta } {p_i(\theta )(1 - p_i(\theta ))~ \left( \frac{{\partial \eta (\theta )}}{{\partial \beta _i }}\right) {\left( \frac{{\partial \eta (\theta )}}{{\partial \beta _i }}\right) ^T}~ \frac{h_i(\theta )}{s}}~ \mathrm{d}\theta . \end{aligned}$$

We now search a locally $\Psi$-optimal design in the set of restricted designs, $\Xi ^g_s$. Like in the unrestricted case (see Sect. 2.2), we can characterize an optimal design using an equivalence theorem. We derive a new equivalence theorem for the case of calibration of multiple items. This equivalence theorem uses again a directional derivative $F_\Psi (h,\theta ,i)$ for design *h* in direction of the one-point measure in $(\theta ,i)$.

For a given design $h^*$, we define the minimum ${\tilde{L}}$ over, the directional derivatives: ${\tilde{L}}(h^*,\theta ) = \min _{i=1,\dots ,n} F_\Psi (h^*,\theta ,i).$ Further, we define for a given sampling proportion *s*

5 $$\begin{aligned} c^* = \arg \max _{c} \left\{ \int _{\Theta } \mathbf{1}_{{\tilde{L}}(h^*,\theta )\le c}~ g(\theta )~\mathrm{d}\theta \le s \right\} , \end{aligned}$$

where $\mathbf{1}_A$ is the indicator function being 1 on a set *A* and 0 otherwise. Let *L* be the at $c^*$ truncated function ${\tilde{L}}$, $L(h^*,\theta ) = \min \{{\tilde{L}}(h^*,\theta ),c^*\}$. When formally defining $F_\Psi (h^*,\theta ,0)=c^*$, we can write

6 $$\begin{aligned} L(h^*,\theta ) = \min _{i=0,1,\dots ,n} F_\Psi (h^*,\theta ,i). \end{aligned}$$

#### Theorem 1

(Equivalence Theorem for Item Calibration) Let $h^* \in \Xi ^g_s$ be a design and $c^*$ and *L* be defined according to (5) and (6) and let $\Psi$ be differentiable. Then: $h^*$ is $\Psi$-optimal in $\Xi ^g_s$ if and only if

7 $$\begin{aligned} F_\Psi (h^*,\theta ,i) = L(h^*,\theta ) \hbox { for } h^* \hbox {-almost all }\,(\theta ,i)\in {\tilde{\chi }}. \end{aligned}$$

We provide a formal proof in “Appendix A”.

The use of this equivalence theorem in applications is: For checking if a given candidate design $h^*$ is optimal, compute and plot the *n* directional derivatives $F_\Psi (h^*,\theta ,i), i=1,\dots ,n$ over $\Theta$. The design is optimal if the sampling is only for items when their directional derivative is smallest and (in case $s<1$) if it is below some constant $c^*$ which separates the regions of sampling (dir. dev. $\le c^*$) from the regions of non-sampling (dir. dev. $\ge c^*$). We will use this theorem for the examples in Sects. 3 and 4.

A consequence of the theorem is that the optimal design usually samples the full available population on ability intervals for a single item. Only if two (or more) directional derivatives coincide on an interval, it can be optimal to sample these two (ore more) items for the same ability interval.

#### Example 3

We focus now specifically on D-optimality, $\Psi \{ M(\xi )\} = - \log \left| {M(\xi )} \right|$ and the two-parameter logistic model (2). Sahm and Schwabe (2000) argue for the logistic regression model, that $F_D(h,\cdot ,i)$, $i\ne 0$, has at most three local extrema for all designs *h*: up to two local minima and one local maximum. Further, $F_D(h,\theta ,i)<2$ for all $\theta \in \Theta$, $\lim _{\theta \rightarrow \pm \infty } F_D(h,\theta ,i)=2$, and $F_D(h,\cdot ,i)$ is a continuous function and not constant on any interval. From these results mentioned by Sahm and Schwabe (2000), we can conclude in the case of a single item to calibrate, $n=1$, that one can search the optimal design specifically in the designs which sample everyone in (at most) two intervals,

8 $$\begin{aligned}{}[\theta _{1L}, \theta _{1U}] \cup [\theta _{2L}, \theta _{2U}] \hbox { with } -\infty< \theta _{1L} \le \theta _{1U} \le \theta _{2L} \le \theta _{2U} < \infty , \end{aligned}$$

and no one outside these intervals. This design has sub-probability density $h_1(\theta ) = g(\theta ) \cdot \mathbf{1}_{[\theta _{1L}, \theta _{1U}] \cup [\theta _{2L}, \theta _{2U}]}(\theta )$. The shape of the directional derivatives discussed above tells specifically for the case $s < 1$ that it is (from a theoretical point of view) never D-optimal to sample the examinees with the lowest and with the highest ability. However, we will see in examples that the optimal design chooses sometimes examinees with very low or very high abilities. If $s=1$, one can show that the D-optimal design allocates examinees with the lowest and the highest ability to the item with the lowest discrimination $a_i$.

### Optimization Algorithm

The optimization algorithm for optimal restricted designs for the one and two item case is presented below. An idea how to extent to larger numbers of items *n* will be visible from the case $n=2$—however, complexity will increase.

#### Optimization Algorithm for $n=1$ Item

For the two-parameter logistic model, a two-interval design is optimal. The standard routine for the construction of locally D-optimal two-interval designs (8) is summarized as:

- Step 1: Choose a starting design $h^0=g \cdot \mathbf{1}_{[\theta ^0_{1L}, \theta ^0_{1U}] \cup [\theta ^0_{2L}, \theta ^0_{2U}]}$ which has density *g* on two intervals $[{\theta ^0 _{1L}},\,{\theta ^0 _{1U}}]$ and $[\,{\theta ^0 _{2L}},\,{\theta ^0 _{2U}}]$ and density 0 otherwise. One may choose the intervals in the starting design around the optimal unrestricted design points which are shown in Sect. 3.
- Step 2: Solve the constrained optimization problem: maximize $\left| {M(\xi )} \right|$ or minimize $- \log \left| {M(\xi )} \right|$ for parameters ${\theta _{1L}},{\theta _{1U}}, {\theta _{2L}}, {\theta _{2U}}$
  - subject to equality constraint $\int \limits _{{\theta _{1L}}}^{{\theta _{1U}}} {g(\theta )\mathrm{d}\theta } + \int \limits _{{\theta _{2L}}}^{{\theta _{2U}}} {g(\theta )\mathrm{d}\theta } = s\,[\hbox {based on }(3)\hbox { and }(4)]$ and
  - subject to inequality constraint ${\theta _{1L}} \le {\theta _{1U}} \le {\theta _{2L}} \le {\theta _{2U}}$.
- Step 3: Finally for assurance, check whether this two-interval design is really D-optimal by computing the directional derivative of the D-criterion and by checking whether the condition in the Equivalence Theorem for Item Calibration is fulfilled.

Note that the equality constraint is not required in Step 1; Step 2 will then ensure it.

#### Optimization Algorithm for $n=2$ Items

If $n=2$ or larger, the number of intervals needed for each item is not necessarily bounded by 2. We start with allowing $K=2$ intervals for each item in any possible order, determine the restricted D-optimal design (given the additional restriction of up to *K* intervals) and check with the Equivalence Theorem for Item Calibration if the design is optimal. If it is not optimal, we repeat these steps for $K+1$. In more detail:

- Step 1: Choose a starting design $\xi ^0$ which has density *g* on *K* intervals
  - for $$1\mathrm{st}$$ item: $${I_{11}}=[{\theta ^0 _{11L}},\,{\theta ^0 _{11U}}], \dots , {I_{1K}}=[{\theta ^0 _{1KL}},\,{\theta ^0 _{1KU}}]$$,
  - for $2\mathrm{nd}$ item: ${I_{21}}=[{\theta ^0 _{21L}},\,{\theta ^0 _{21U}}], \dots , {I_{2K}}=[{\theta ^0 _{2KL}},\,{\theta ^0 _{2KU}}]$ and density 0 otherwise.
- Step 2: Solve the constrained optimization problem: maximize $\left| {M(\xi )} \right| = \left| {M_1(\xi _1 )} \right| \cdot \left| {M_2(\xi _2 )} \right|$ or minimize
  9 $$\begin{aligned} - \log \left| {{M_1}(\xi _1 )} \right| - \log \left| {{M_2}(\xi _2 )} \right| \end{aligned}$$
  - subject to equality constraint $\sum \limits _{r = 1}^n {\sum \limits _{t = 1}^K {\int \limits _{{I_{rt}}}^{} {g(\theta )\mathrm{d}\theta } = s} } {}$ and
  - subject to inequality constraint ${I_{11}}\dots {I_{1K}}{I_{21}}\dots {I_{2K}}\Leftrightarrow \theta _{11L}\le \theta _{11U} \le \dots \le \theta _{12L} \le \theta _{12U} \le \theta _{21L}\le \theta _{21U} \le \dots \le \theta _{22L} \le \theta _{22U}$.
  Similarly we check other possible ordering of intervals. For $K=2$, we have six inequalities constraints ${I_{11}}{I_{12}}{I_{21}}{I_{22}}$, ${I_{11}}{I_{21}}{I_{12}}{I_{22}}$, ${I_{11}}{I_{21}}{I_{22}}{I_{12}}$, ${{I_{21}}{I_{22}}I_{11}}{I_{12}}$, ${{I_{21}}I_{11}}{I_{22}}{I_{12}}$, ${{I_{21}}I_{11}}{I_{12}}{I_{22}}$. We select the inequity constraint which gives minimum value in (9).
- Step 3: Finally for assurance, it is essential to check whether this *K*-interval design is really D-optimal by computing the directional derivative of the D-criterion and by checking whether the condition in the Equivalence Theorem for Item Calibration is fulfilled. If the design is not optimal, set $K=K+1$ and go to Step 1.

The number of inequalities constraints is generally $$\left( {\begin{array}{c}2K\\ K\end{array}}\right) $$. In the computations we made for the examples in Sect. 4, it was sufficient to use $K=2$ and $K=3$.

Since it is allowed that two or more interval boundaries coincide, the designs which need less than *K* intervals are special cases of a *K*-interval design. Hence, when increasing *K*, (9) cannot increase; it is decreasing until the right *K* is found and would then be constant for larger *K*. If some interval boundaries coincide in the determined optimal design, we can finally reduce the number of intervals of it.

The constrained optimization problem in our algorithm was solved by using the R-package nloptr (Borchers, 2013) with Sequential (least-squares) Quadratic Programming (SQP) algorithm. We use the algorithm for the examples presented in Sects. 3 and 4. The number of iterations of the SQP algorithm can vary considerably for each case, but a final solution is obtained in all cases very quickly within one minute.

### Relative Efficiency of Designs

The relative D-efficiency ($\mathrm{RE}_\mathrm{D}$) of a design $h^{(1)}$ compared to another design $h^{(2)}$ is

10 $$\begin{aligned} {\mathrm{RE}_\mathrm{D}} = \mathrm{RE}_\mathrm{D}(h^{(1)},h^{(2)})= {\left[ {\frac{{|M({h^{(1)}})|}}{{|M({h^{(2)}})|}}} \right] ^{\frac{1}{2n}}} = {\left[ {\frac{{\prod \nolimits _{i = 1}^n {|{M_i}\left( {h_{i}^{(1)}}\right) |} }}{{\prod \nolimits _{i = 1}^n {|{M_i}\left( h_i^{(2)}\right) |} }}} \right] ^{\frac{1}{2n}}} \end{aligned}$$

where 2*n* is the number of parameters, see Berger and Wong (2009). An $\mathrm{RE}_\mathrm{D}$-value less than 1 indicates that design $h^{(2)}$ is better than design $h^{(1)}$ in terms of D-optimality. In terms of sample size, this means that design $h^{(1)}$ approximately needs $(\mathrm{RE}_\mathrm{D}^{ - 1} - 1)*100\%$ more examinees to be as efficient as $h^{(2)}$.

We could assign the items randomly irrespectively of the ability such that each examinee has probability *s* / *n* to calibrate a specific item. This so-called random design has densities $h_i^r=sg/n$ for $i=1,\dots ,n$. In the examples, we will be interested, e.g., in the relative efficiency $\mathrm{RE}_\mathrm{D}(h^r,h^*)$ of the random design compared to the D-optimal restricted design $h^*$. Many researchers have compared optimal design efficiency with a random design in item calibration studies, see e.g., Buyske (2005). We consider a random design as a benchmark for comparison.

Another important design for comparison is the symmetric design. It divides first the sample proportion *s* equally to all, say *m*, unrestricted design points and a proportion *s* / *m* should be sampled around a design point $\theta ^*$. A value *d* is computed such that the desired proportion *s* / *m* of examinees is in the symmetric interval $(\theta ^*-d,\theta ^*+d)$, i.e., $\int _{\theta ^*-d}^{\theta ^*+d} g(x)~\mathrm{d}x=s/m$. The symmetric design is only well defined as long as the intervals from the design points do not overlap.

## Results for Calibration of One Item

We consider now three new items to calibrate for our item bank. Assuming a two-parameter logistic model (2), the best guess items parameters $a_i$ and $b_i$ of these items are:

- Item 1: Discrimination $a_1=1$, difficulty $b_1=0.5$;
- Item 2: Discrimination $a_2=1.5$, difficulty $b_2=-1.2$;
- Item 3: Discrimination $a_3=1.6$, difficulty $b_3=2$.

Usually, the number of items to calibrate is larger than the number which can be allocated to a single examinee. Imagine, e.g., a situation where we have ten times more new items than can be calibrated by an examinee. Focusing first on a specific new item, it should therefore be assigned to approximately 10% of the examinees. In this section, we illustrate scenarios where we choose a proportion $s<1$ of examinees to calibrate a single item, $n=1$. We ignore in this section that items cannot be treated separately as they might need the same examinees for calibration. In Sects. 4 and 5, we will calibrate then several items simultaneously.

Abdelbasit and Plackett (1983) showed that for the two-parameter logistic model the locally D-optimal design for a single item with best guess values *a* and *b* for discrimination and difficulty has two distinct equally weighted design points or ability levels ${\theta } = b \pm \frac{{1.543}}{a}$. The corresponding probabilities of correctly answering the question at these points, ${\theta _1}$ and ${\theta _2}$ say, are $p({\theta _1}) = 0.176$ and $p({\theta _2}) = 0.824$. This means that the unrestricted locally D-optimal design recommends to choose half of the examinees for calibration with ability $\theta _1= b\,\mathrm{{ - }}\frac{{1.543}}{a}$ and half with ability $\theta _2= b\,+\frac{{1.543}}{a}$.

We assume in the examples in Sects. 3 and 4 that the examinees in the computerized test have standard normal distributed abilities. We compute locally D-optimal restricted designs with restriction $g(\theta ) = \frac{1}{{\sqrt{2\pi } }}{e^{( - \frac{{{\theta ^2}}}{2})}}$. However, the method including the Equivalence Theorem in Sect. 2.3 is valid even if another assumption for the abilities is preferred. Since we compute here locally optimal designs, we have made investigations in the supplementary materials where we see robustness as long as the parameters are not severely misspecified.

### Calibration of Item 1

**Fig. 1:**
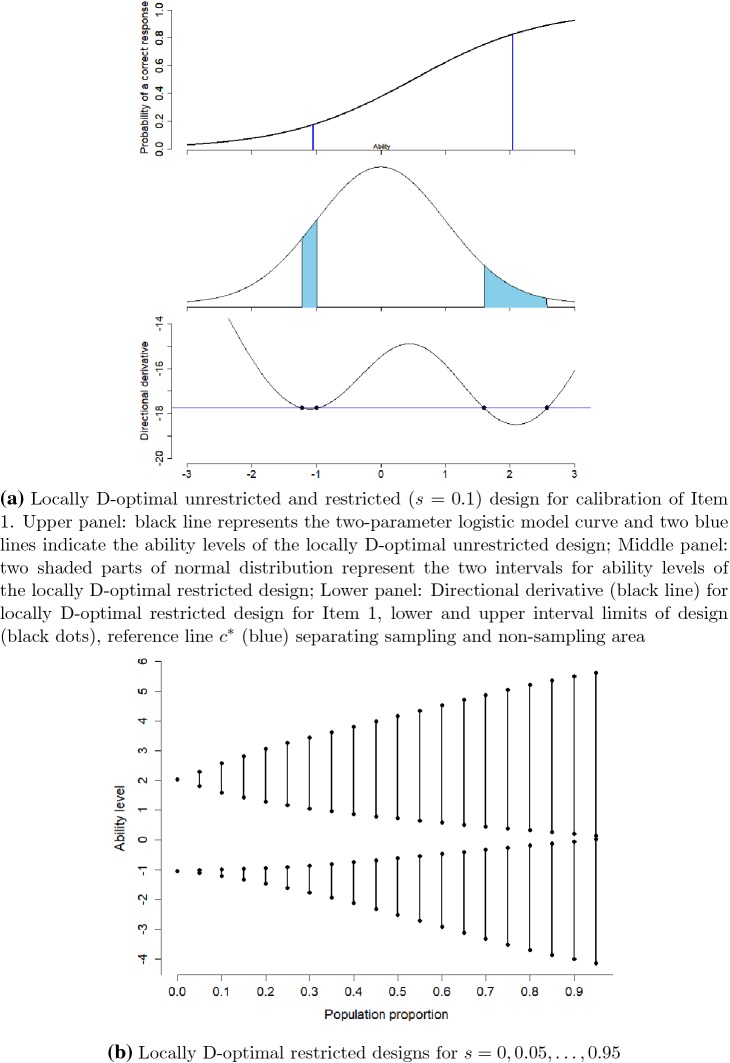
Locally D-optimal restricted designs for Item 1 (Color figure online).

In the first scenario, we consider an item with best guess for the difficulty parameter $b=0.5$ and item discrimination parameter $a=1$ (Item 1). We want to select 10% of the population of examinees to calibrate this item in the item bank. The unrestricted optimal design recommends to sample 5% examinees with ability level $0.5-\frac{1.543}{1}=-1.043$ and 5% with $0.5+\frac{1.543}{1}=2.043$. The best guess probability for correct response and the optimal unrestricted design points are shown in the upper panel of Fig. 1a.

**Fig. 2:**
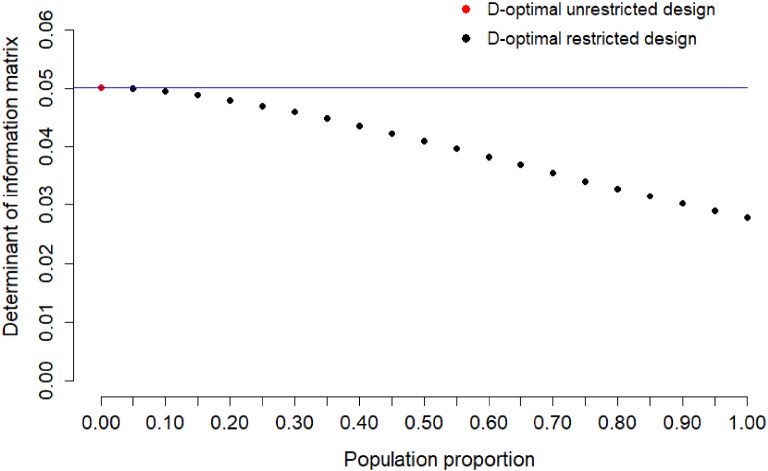
Determinant of information matrix of locally D-optimal restricted design for calibration of Item 1 for sample proportion $s=0,0.05,\dots ,0.95,1$. The blue line indicates the maximum value of determinant of the information matrix of two-point unrestricted design (Color figure online).

It is hard to select a sample of examinees with these specific ability levels as there might be no such examinees available or we have a limited number of examinees with these ability levels. We sample instead the examinees from the available distribution in an optimal way using the techniques described in Sect. 2.3. For the restricted optimal design, we assume that the population of examinees has a standard normal ability distribution and we sample a proportion $s=0.1$ of this population. The calculated optimal restricted design recommends to sample 5% examinees from the population with ability level between (− 1.215, $-0.984$) and 5% between (1.600, 2.577), see the middle panel of Fig. 1a. The intervals are not equal in length: We have limited available examinees around the high unrestricted ability level 2.043 compared to the low level $-1.043$. So we need a longer interval around the unrestricted ability level 2.043 to select 5% examinees of population. The intervals are also asymmetrical around the unrestricted design points and extend more toward the extreme abilities since less examinees are available there.

The directional derivative for this two-interval design is shown in the lower panel of Fig. 1a with black line and interval limits are marked on it with red dots. Since these four points of the two-interval design are on one blue reference line and the intervals of the population sample have directional derivative below the reference line, the Equivalence Theorem for Item Calibration described in Sect. 2.3 confirms the optimality of this two-interval design (the blue reference line corresponds to $c^*$ in the theorem).

We computed the optimal restricted design for other sample proportions than $s=0.1$. We show the results in Fig. 1b for $s=0, 0.05, 0.1,\dots ,0.95$, where $s=0$ is the limiting case of unrestricted optimal design. We see there that we still have a two-interval design if we want to sample 95% of the population. This two-interval design becomes one interval if we sample 96% of the population. Figure 2 shows the determinant of the information matrix of the locally D-optimal restricted design for Item 1 for sample proportion $s=0,0.05,\dots ,0.95,1$. The case $s=0$ corresponds to the locally D-optimal unrestricted design, $s=1$ to the random design. The loss of information for Item 1 is moderate if the population proportion is between 0.0 and 0.2.

### Calibration of Item 2

**Fig. 3:**
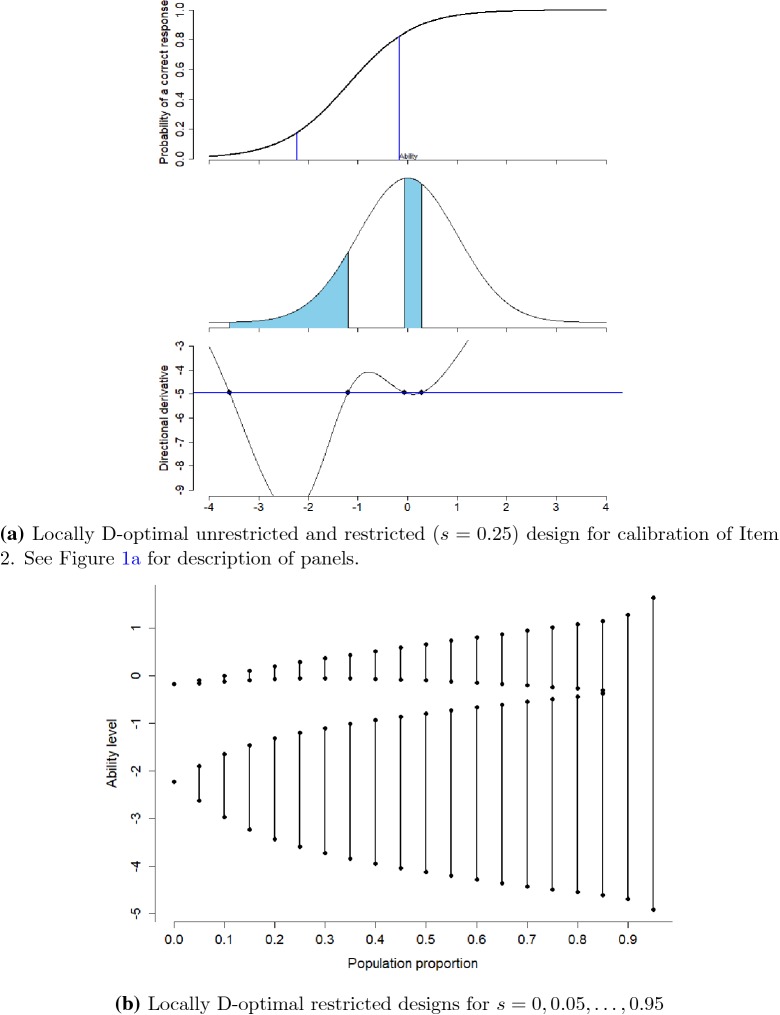
Locally D-optimal restricted designs for Item 2.

**Fig. 4:**
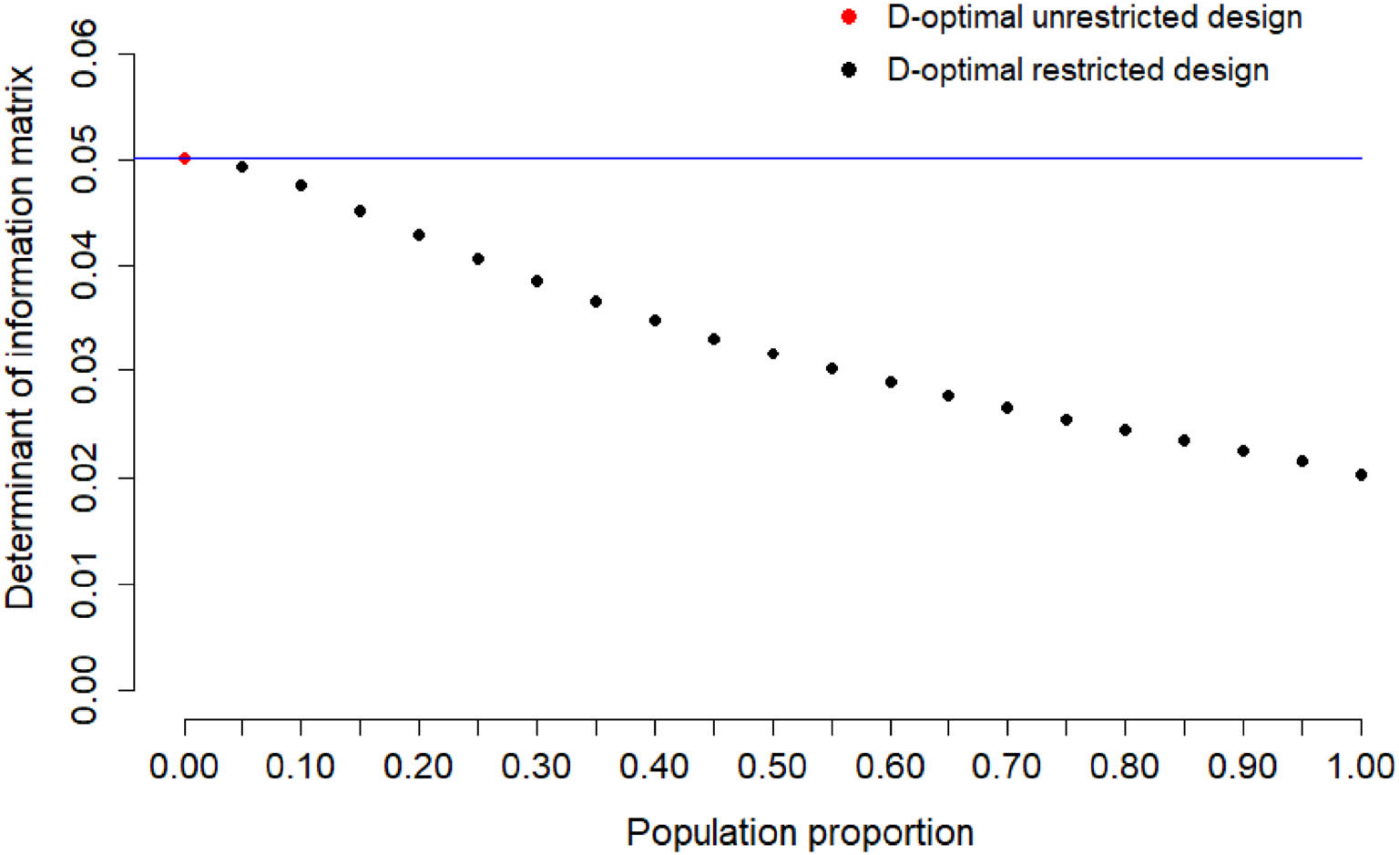
Determinant of information matrix of locally D-optimal restricted design for calibration of Item 2 for sample proportion $s=0,0.05,\dots ,0.95,1$. The blue line indicates the maximum value of determinant of the information matrix of two-point unrestricted design (Color figure online).

Now we discuss another scenario where we want to calibrate Item 2 with best guess for difficulty parameter $b=-1.2$ and discrimination parameter $a=1.5$. To calibrate this item, we want to sample 25% of examinees population ($s=0.25$). The unrestricted design recommends to sample 12.5% of examinees at each of the ability levels $-2.229$ and $-0.171$. Again we use restricted optimal design because of unavailability or limited available examinees with these specific ability levels. To calibrate this item, we sample 11.48% from the population of examinees between ability levels ($-3.592$, $-1.200$) and 13.52% between ($-0.061$, 0.282), see Fig. 3a with design and directional derivative plot. In this scenario, the selected sample proportion of the population for the two intervals is not equal. Another remarkable fact is that the upper interval not contains the upper value of the optimal unrestricted design. So sampling around this optimal unrestricted design point definitely produces a non-optimal restricted design. Since around $\theta =0$ more examinees are available compared to ability level $-2.229$, the upper interval has a shorter length compared to the lower. However in this case, the symmetric design needs only 1.98% more examinees to have the same efficiency than the restricted D-optimal design, i.e., it is an efficient design; the random design needs 45.45% more examinees. When investigating other sample proportions *s*, we see that the optimal two-interval design will become a one-interval design if we want to sample a proportion of 90% or more of the population to calibrate the item. Results for other *s* are presented in Fig. 3b. For Item 2, we show the determinant of the information matrix of the locally D-optimal restricted design for different sample proportions in Fig. 4. The loss of information for Item 2 is moderate with population proportions between 0.0 and 0.2.

### Calibration of Item 3

**Fig. 5:**
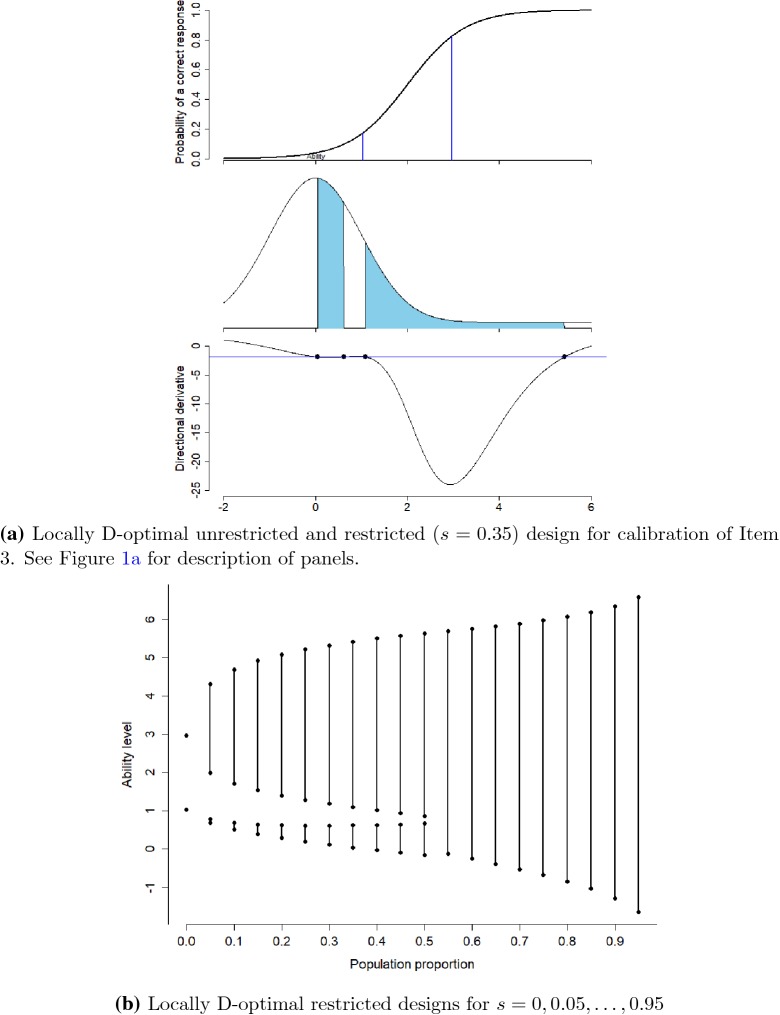
Locally D-optimal restricted designs for Item 3.

In the third scenario, we want to sample 35% of the examinees population ($s=0.35$) in order to calibrate Item 3 with best guess for difficulty parameter $b=2$ and discrimination parameter $a=1.6$. The unrestricted optimal design recommends to choose 17.5% sample proportion of the population at each of the ability levels 1.035 and 2.965. The restricted optimal design samples 21.23% from the population of examinees between ability levels (0.043, 0.611) and 13.76% between (1.091, 5.417), see Fig. 5a with design and directional derivative plot. Both intervals have unequal length and different sample proportions of the examinees population. The lower limit of the upper interval is quite close to the lower point of the optimal unrestricted design. This seems reasonable as limited examinees are available around the high ability level 2.965. So to select the examinees this lower limit of the upper interval moves toward the left as more examinees are available to this side. To counter this, the lower interval is quite below from the lower point of the optimal unrestricted design. Similarly as for Item 2, the lower interval does here not contain the lower unrestricted design point. This effect happens for items with difficulty *b* not in the center of the ability distribution. The value of difficulty where this effect starts depends on the discrimination *a*. In the supplementary materials, we provide figures showing combinations of *a* and *b* where an unrestricted design point is not contained in the restricted optimal design. 

**Fig. 6:**
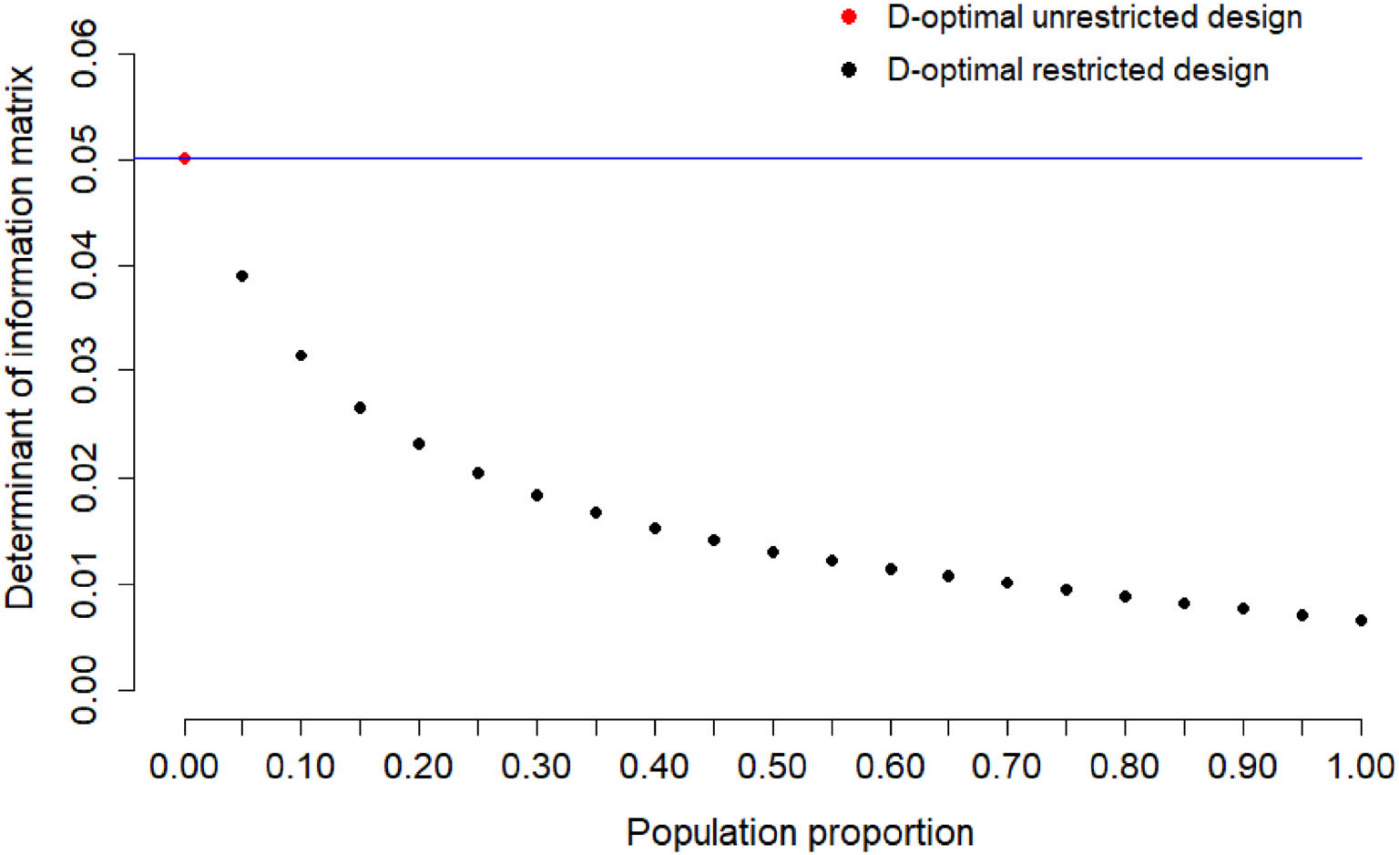
Determinant of information matrix of locally D-optimal restricted design for calibration of Item 3 for sample proportion $s=0,0.05,\dots ,0.95,1$. The blue line indicates the maximum value of determinant of the information matrix of two-point unrestricted design (Color figure online).

In terms of sample size, the random design needs 59.66% more examinees to have the same efficiency as a restricted D-optimal design. If we would try to select examinees symmetrically around the points of the unrestricted design, we have the problem that the intervals around the two design points overlap, i.e., the symmetric design is not usable directly. For sampling proportion $s=0.2$, the symmetric design has no overlapping intervals; in that case, the symmetric design needs 12.95% more examinees to have the same efficiency than the restricted D-optimal design. Investigating other sample proportions *s*, the optimal two-interval design will become a one-interval design if we sample a proportion of 55% or more of the population, see Fig. 5b. Figure 6 indicates that the loss of information is considerable for Item 3 if the population proportion is between 0.0 and 0.2.

**Table 1:** Relative efficiency of random design versus D-optimal restricted design.

Proportion (%)	Item 1		Item 2		Item 3	
	$\mathrm{RE}_\mathrm{D}$	$\mathrm{RE}_\mathrm{SS} (\%)$	$\mathrm{RE}_\mathrm{D}$	$\mathrm{RE}_\mathrm{SS} (\%)$	$\mathrm{RE}_\mathrm{D}$	$\mathrm{RE}_\mathrm{SS} (\%)$
0	0.7451	34.2076	0.6360	57.2429	0.3616	176.5380
5	0.7463	33.9931	0.6407	56.0771	0.4094	144.2796
10	0.7498	33.3776	0.6531	53.1182	0.4562	119.2079
15	0.7551	32.4376	0.6695	49.3729	0.4964	101.4377
20	0.7618	31.2638	0.6875	45.4549	0.5325	87.7791
25	0.7697	29.9238	0.7062	41.6082	0.5658	76.7462
30	0.7785	28.4552	0.7251	37.9183	0.5969	67.5348
35	0.7882	26.8741	0.7440	34.4095	0.6263	59.6613
40	0.7988	25.1845	0.7629	31.0821	0.6544	52.8130
45	0.8105	23.3856	0.7817	27.9273	0.6813	46.7767
50	0.8232	21.4783	0.8004	24.9334	0.7072	41.4018
55	0.8370	19.4692	0.8191	22.0882	0.7329	36.4384
60	0.8520	17.3720	0.8377	19.3804	0.7563	32.2257
65	0.8680	15.2055	0.8562	16.8001	0.7807	28.0906
70	0.8850	12.9922	0.8746	14.3393	0.8063	24.0296
75	0.9029	10.7552	0.8929	11.9919	0.8331	20.0334
80	0.9215	8.5176	0.9111	9.7539	0.8614	16.0870
85	0.9407	6.3018	0.9292	7.6245	0.8915	12.1685
90	0.9603	4.1296	0.9472	5.5690	0.9238	8.2444
95	0.9802	2.0222	0.9692	3.1792	0.9592	4.2536

Relative efficiency $\mathrm{RE}_\mathrm{D}$ versus D-optimal restricted design for calibration of one item. $\mathrm{RE}_\mathrm{SS}$ indicates sample size gain when using optimal restricted design instead of symmetric design.

**Table 2:** Relative efficiency of symmetric design versus D-optimal restricted design.

Proportion (%)	Item 1		Proportion (%)	Item 2	
	$\mathrm{RE}_\mathrm{D}$	$\mathrm{RE}_\mathrm{SS} (\%)$		$\mathrm{RE}_\mathrm{D}$	$\mathrm{RE}_\mathrm{SS} (\%)$
0	1.0000	0.0000	0	1.0000	0.0000
5	0.9999	0.0063	5	0.9993	0.0661
10	0.9994	0.0646	10	0.9954	0.4620
15	0.9980	0.1990	15	0.9887	1.1451
20	0.9961	0.3953	20	0.9806	1.9777
25	0.9937	0.6335	25	0.9722	2.8640
30	0.9911	0.8937	30	0.9639	3.7426
35	0.9886	1.1562	35	0.9563	4.5690
40	0.9862	1.4028	40	0.9496	5.3106
45	0.9841	1.6155	45	0.9439	5.9429
50	0.9825	1.7809	50	0.9394	6.4461
55	0.9814	1.8914	55	0.9363	6.8045
60	0.9809	1.9450			
65	0.9809	1.9463	Proportion (%)	Item 3	
70	0.9813	1.9018		$\mathrm{RE}_\mathrm{D}$	$\mathrm{RE}_\mathrm{SS}$ (%)
75	0.9821	1.8195	0	1.0000	0.0000
80	0.9832	1.7072	5	0.9633	3.8065
85	0.9845	1.5726	10	0.9252	8.0835
90	0.9860	1.4229	15	0.8998	11.1370
95	0.9875	1.2639	20	0.8853	12.9498

Relative efficiency $\mathrm{RE}_\mathrm{D}$ versus D-optimal restricted design for calibration of one item. $\mathrm{RE}_\mathrm{SS}$ indicates sample size gain when using optimal restricted design instead of symmetric design.

**Table 3:** Relative efficiency $\mathrm{RE}_\mathrm{D}$ versus D-optimal restricted design for calibration of two or more items.

Proportion (%)	Item 1, 2		Item 1, 3		Item 2, 3	
	$\mathrm{RE}_\mathrm{D}$	$\mathrm{RE}_\mathrm{SS} (\%)$	$\mathrm{RE}_\mathrm{D}$	$\mathrm{RE}_\mathrm{SS} (\%)$	$\mathrm{RE}_\mathrm{D}$	$\mathrm{RE}_\mathrm{SS} (\%)$
(a) Relative efficiency of random design versus D-optimal restricted design						
0	0.6884	45.2694	0.5191	92.6486	0.4796	108.5273
10	0.6915	44.6164	0.5528	80.8993	0.5113	95.5664
20	0.6997	42.9275	0.5896	69.6201	0.5444	83.6903
30	0.7107	40.7010	0.6226	60.6242	0.5748	73.9856
40	0.7238	38.1549	0.6531	53.1150	0.6032	65.7839
50	0.7404	35.0594	0.6821	46.5996	0.6304	58.6238
60	0.7597	31.6309	0.7104	40.7728	0.6585	51.8552
70	0.7810	28.0483	0.7399	35.1514	0.6871	45.5363
80	0.8037	24.4270	0.7715	29.6245	0.7137	40.1228
90	0.8275	20.8469	0.8048	24.2492	0.7392	35.2876
100	0.8525	17.2983	0.8398	19.0805	0.7665	30.4547

Proportion (%)	Item 1, 2		Item 2, 3			
	$\mathrm{RE}_\mathrm{D}$	$\mathrm{RE}_\mathrm{SS} (\%)$	$\mathrm{RE}_\mathrm{D}$	$\mathrm{RE}_\mathrm{SS} (\%)$		
(b) Relative efficiency of symmetric design versus D-optimal restricted design						
0	1.0000	0.0000	1.0000	0.0000		
10	0.9996	0.0370	0.9796	2.0784		
20	0.9972	0.2772	0.9571	4.4781		
30	0.9930	0.7070	0.9404	6.3430		
40	0.9885	1.1670	0.9289	7.6589		

$\mathrm{RE}_\mathrm{SS}$ indicates sample size gain when using optimal restricted design instead of symmetric design.

### Relative Efficiency of the Optimal Design

Table 1 shows the relative efficiency of the random design versus the D-optimal restricted design for each of the three items. The D-optimal restricted design is generally very efficient compared to the random design gaining up to 34% sample size for Item 1, up to 56% for Item 2, and 144% for Item 3 for giving the same precision of estimates. Additionally for the D-optimal restricted and random designs, we provide figures with the determinants of the information matrices for the three items in the supplementary materials. Table 2 shows efficiencies for the symmetric design. For Item 1 which has a difficulty close to the mean ability of the population, the symmetric design is quite efficient needing only up to 1.95% more examinees compared to the restricted D-optimal design. For Item 2 and 3, the intervals of the symmetric design would overlap for larger *s*; therefore, the design is only possible for $s\le 0.55$ and $s\le 0.2$, respectively. For these items having one unrestricted optimal design point where only few examinees are available, there is a higher sample size gain of the optimal compared to the symmetric design for some *s* (up to 6.80% for Item 2 and 12.95% for Item 3).

## Results for Calibration of Two or More Items

We now present scenarios for calibration of two items. We start with briefly mentioning the locally D-optimal unrestricted design. One can show that it is D-optimal to sample exactly half of the examinees for each of the two items. Within each item, the one-item optimal design mentioned in Sect. 3 is the best choice. This means, the locally D-optimal design for calibration of two items is to sample 25% of the examinees with ability levels $\theta = b_1 \pm \frac{1.543}{a_1}$ for Item 1 and 25% of the examinees with ability levels $\theta = b_2 \pm \frac{1.543}{a_2}$ for Item 2.

We compute now locally D-optimal restricted designs assuming that the examinees participating in the computerized test have standard normal distributed abilities. We use Item 1, 2, and 3 from Sect. 3 and compute the optimal design when at least two of these three items should be calibrated simultaneously.

In a first case (Sect. 4.1), the optimal designs for each of the two items are not overlapping. In more challenging cases (see Sects. 4.2 and 4.3), it can be seen that some examinees would be needed for both items – they compete with each other. Then, the optimal design will determine the best allocation to either of the items. The result can be a two-interval solution for both items (Sect. 4.2); in this case, the algorithm in Sect. 2.4.2 found the optimal design using $K=2$. In Sect. 4.3, $K=3$ intervals were needed for each item. Finally, we compute optimal designs when all examinees are sampled ($s=1$; Sects. 4.4 and 4.5). In Table 3a, the relative efficiencies are calculated for the random design. Considerable sample size gains exist in all cases (17.30% to 95.57%). Table 3b shows efficiencies for the symmetric design in cases where intervals do not overlap. We see that in many cases including all cases for calibrating Item 1 and 3, we cannot apply the symmetric design directly due to overlapping.

### Calibration for Non-competing Items

In this first situation, we consider Item 1 ($a=1$, $b=0.5$) and Item 2 ($a=1.5$, $b=-1.2$) for calibration. We are interested to sample 10% population of examinees to calibrate these two items in the item bank.

**Fig. 7:**
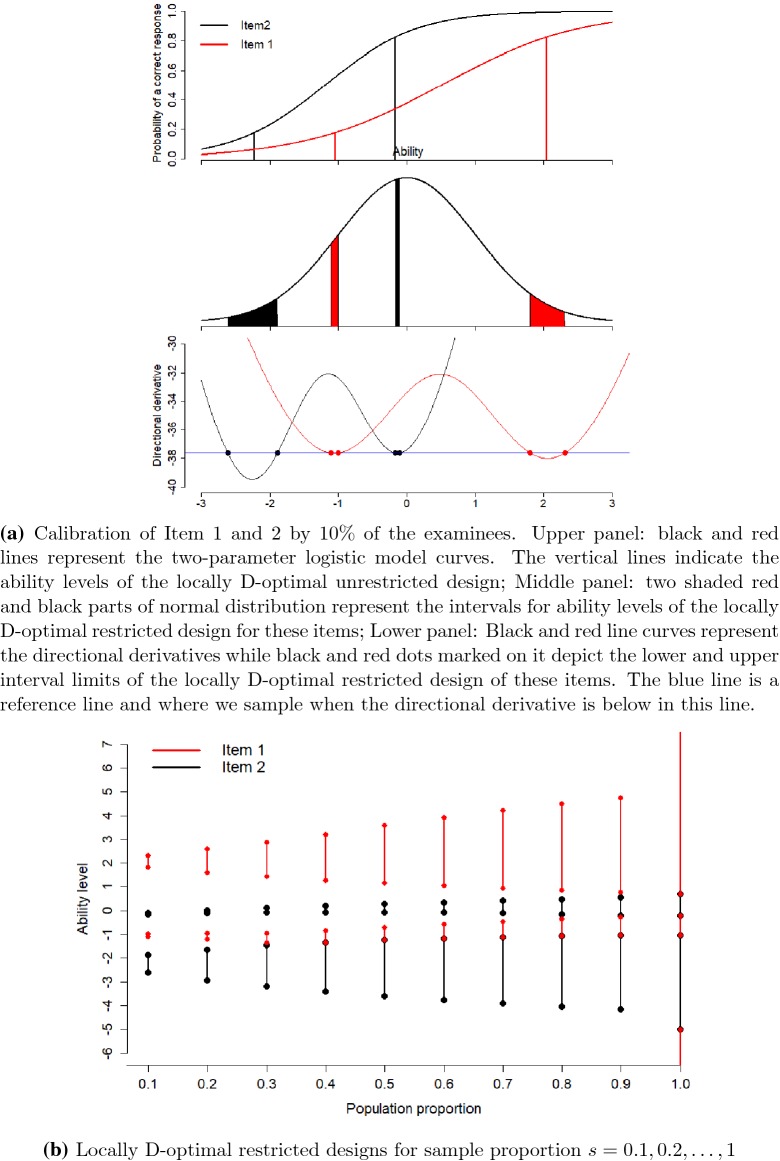
Locally D-optimal restricted designs for simultaneous calibration of Item 1 and 2.

The unrestricted optimal design suggests to sample 2.5% examinees at each ability levels $-1.043$, 2.043 (for Item 1) and $-2.229, -0.171$ (for Item 2). Since it is in practice hard to sample the examinees at these specific ability levels due to unavailability or limited availability of number of examinees, we use restricted optimal design to sample the examinees between some intervals of ability levels in an optimal way. The unrestricted optimal design recommends us to sample 2.52% and 2.51% examinees from the population between ability levels ($-1.114$, $-1.004$) and (1.804, 2.308), respectively, for Item 1. For Item 2, it suggests to choose 2.47% and 2.50% of the examinees between ability levels ($-2.617$, $-1.893$) and ($-0.167$, $-0.103$), see Fig. 7a. The directional derivative plot in the lower panel of Fig. 7a confirms that the design with these intervals limits is optimal: The blue reference line (corresponding to value $c^*$ in the Equivalence Theorem for Item Calibration) separates the sampling regions from the non-sampling regions. Further, the sampling to item $i, i=1,2,$ corresponds to the region where the respective item has the smallest directional derivative. We show optimal designs for other values of $s \in \{0.1, 0.2, \dots , 1\}$ in Fig. 7b.

### Calibration for Competing Items

**Fig. 8:**
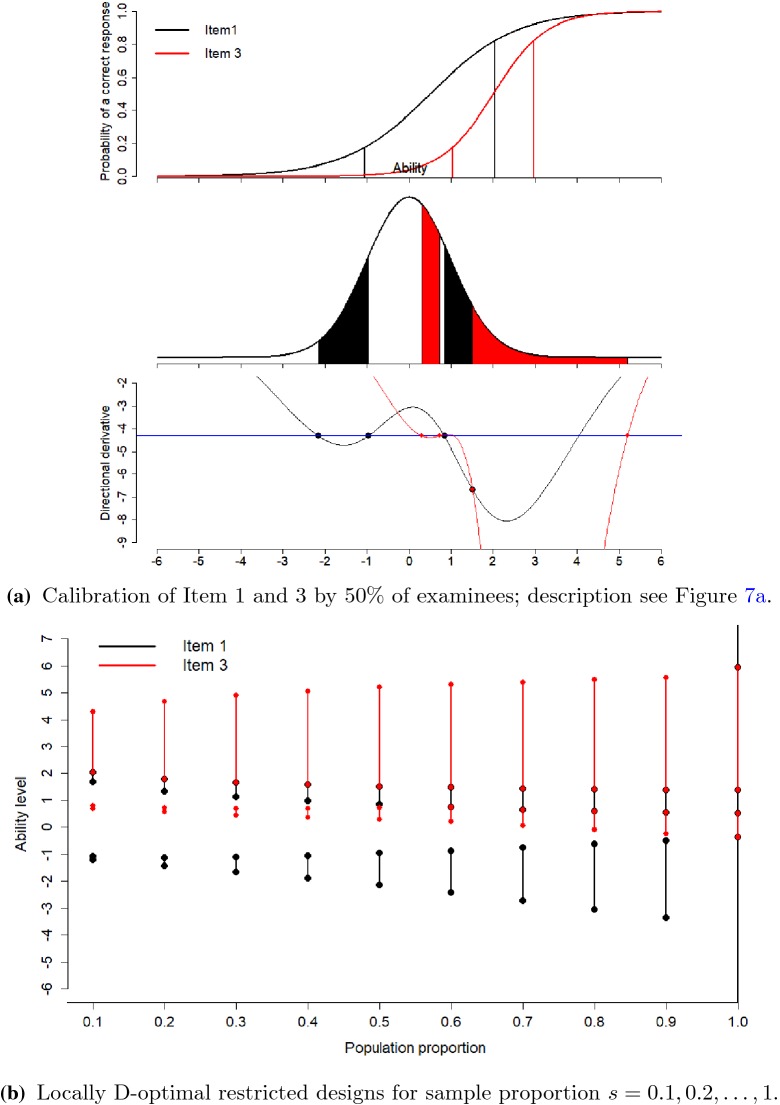
Locally D-optimal restricted designs for simultaneous calibration of Item 1 and 3.

In this case, we want to select a sample of 50% examinees from the population in order to calibrate Item 1 ($a=1$, $b=0.5$) and Item 3 ($a=1.6$, $b=2$) in the item bank. The unrestricted optimal design would select 12.5% examinees each at the ability levels $-1.043$, 2.043 for Item 1 and 12.5% examinees at each ability levels 1.035, 2.965 for Item 3. Selecting examinees around the unrestricted design points in a naive manner faces the problem that there are only few examinees around the ability levels 2.043 and 2.965. The restricted design recommends us to choose 15.1% and 13.6% of the population of examinees on the ability intervals ($-2.158$, $-0.967$) and (0.836, 1.511) for Item 1 and choose 14.7% and 6.5% of the examinees for Item 3 on the intervals (0.299, 0.721) and (1.511, 5.197), see Fig. 8a. The directional derivative plot in the lower panel confirms based on the Equivalence Theorem for Item Calibration that this restricted optimal design is optimal. In each region, the item with the lowest directional derivative is sampled. The two upper intervals follow directly after each other with boundary point 1.511. This shows that the two items are competing for examinees around this ability $\theta =1.511$. The directional derivative of both items is equal at this point. Examinees with such $\theta$ would be good for both items since both directional derivatives are well below the reference line, but in order to maximize the overall information, this cut-point was determined. Figure 8b shows optimal designs for other values of $s \in \{0.1, 0.2, \dots , 1\}$.

### Calibration for Items with Several Intervals

**Fig. 9:**
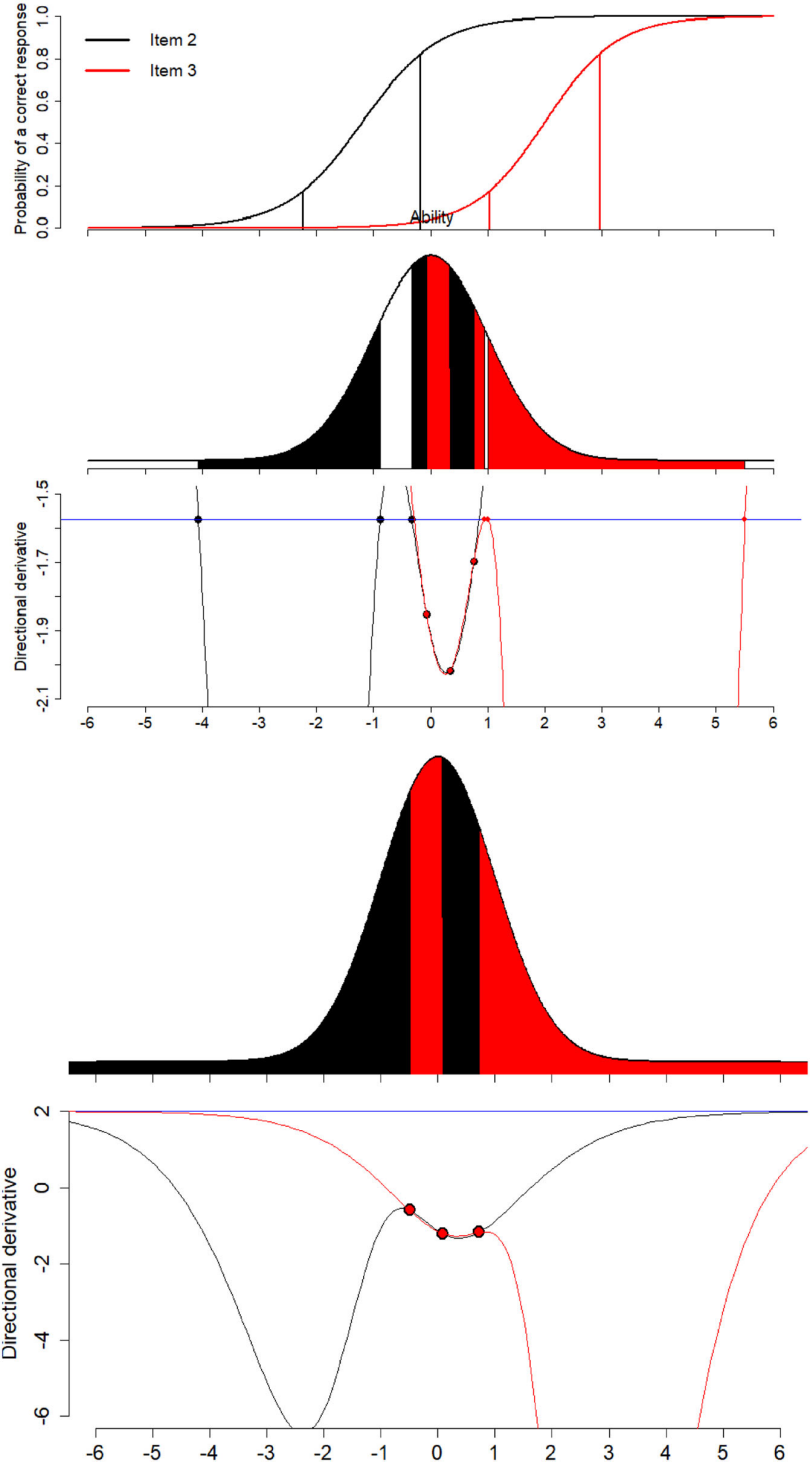
Calibration of Item 2 and 3 using 80% of examinees and the whole population of examinees, see Fig. 7a.

**Fig. 10:**
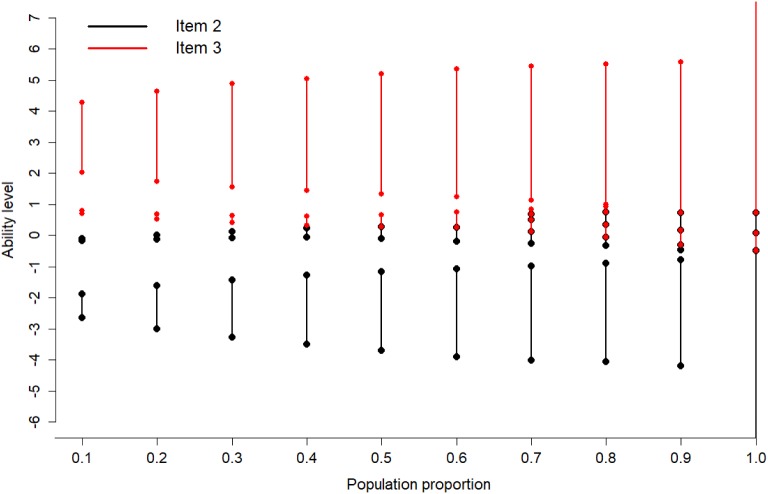
Locally D-optimal restricted designs for simultaneous calibration of Item 2 and 3 for sample proportion $s=0.1,0.2,\dots ,1$.

Now in this situation we want to choose a sample of 80% examinees from the population to calibrate Item 2 ($a=1.5$, $b=-1.2$) and Item 3 ($a=1.6$, $b=2$) in the item bank. The unrestricted design recommends us to choose 20% examinees each with abilities $-2.229$, $-0.171$ for Item 2 and 1.035, 2.965 for Item 3. The restricted optimal design suggests us to select 18.78%, 10.14% and 14.29% of the population of examinees on the ability intervals ($-4.069$, $-0.886$), ($-0.329$, $-0.069$) and (0.338, 0.757), respectively, for Item 2. It also recommends to choose 16.01%, 5.05% and 15.72% of examinees from the population on the ability intervals ($-0.069$, 0.338), (0.757, 0.938) and (1.006, 5.508) for Item 3, see Fig. 9. The directional derivative plot in the lower panel of Fig. 9 shows together with the Equivalence Theorem for Item Calibration that this design is optimal for the selection of examinees: We select examinees on intervals below the blue line for Item 2 or 3 depending on which item’s directional derivative is smallest in these intervals. In contrast to the preceding example, the competition between the items leads here to the need of three intervals for each item. Note that in the region from $\theta =-0.3$ to 0.9, the two directional derivatives are quite close but do not exactly coincide—the minimum is unique except for the crossing points. Optimal designs for other values of $s \in \{0.1, 0.2, \dots , 1\}$ are presented in Fig. 10.

### Calibration of Two Items Using the Whole Population

Now we want to select all the available examinees in order to calibrate Item 2 and 3 in the item bank. The optimal unrestricted design suggests to choose 25% each of all available examinees at the ability levels $-2.229$ & $-0.171$ for Item 2 and 25% each for Item 3 at the ability levels 1.035 & 2.965. When we use restricted optimal design, we should choose 30.98% of the examinees on the ability interval ($-\infty$, $-0.496$) and 23.28% on (0.081, 0.723) for Item 2. For Item 3, it suggests to choose 22.27% examinees on ($-0.496$, 0.081) and 23.47% on (0.723, $\infty$). (With an exact computation, the last interval is obtained to be (0.723, 10); examinees with higher ability should receive Item 2. However, the probability for an ability $\ge 10$ is basically 0.) The directional derivative in the third panel of Fig. 9 shows that this design is optimal for selection of examinees: We choose examinees for Item 2 or 3 whenever the directional derivative is smallest. The random design requires 30.45% more examinees to be as efficient as the locally D-optimal restricted design.

### Calibration of All Three Items Using the Whole Population

**Fig. 11:**
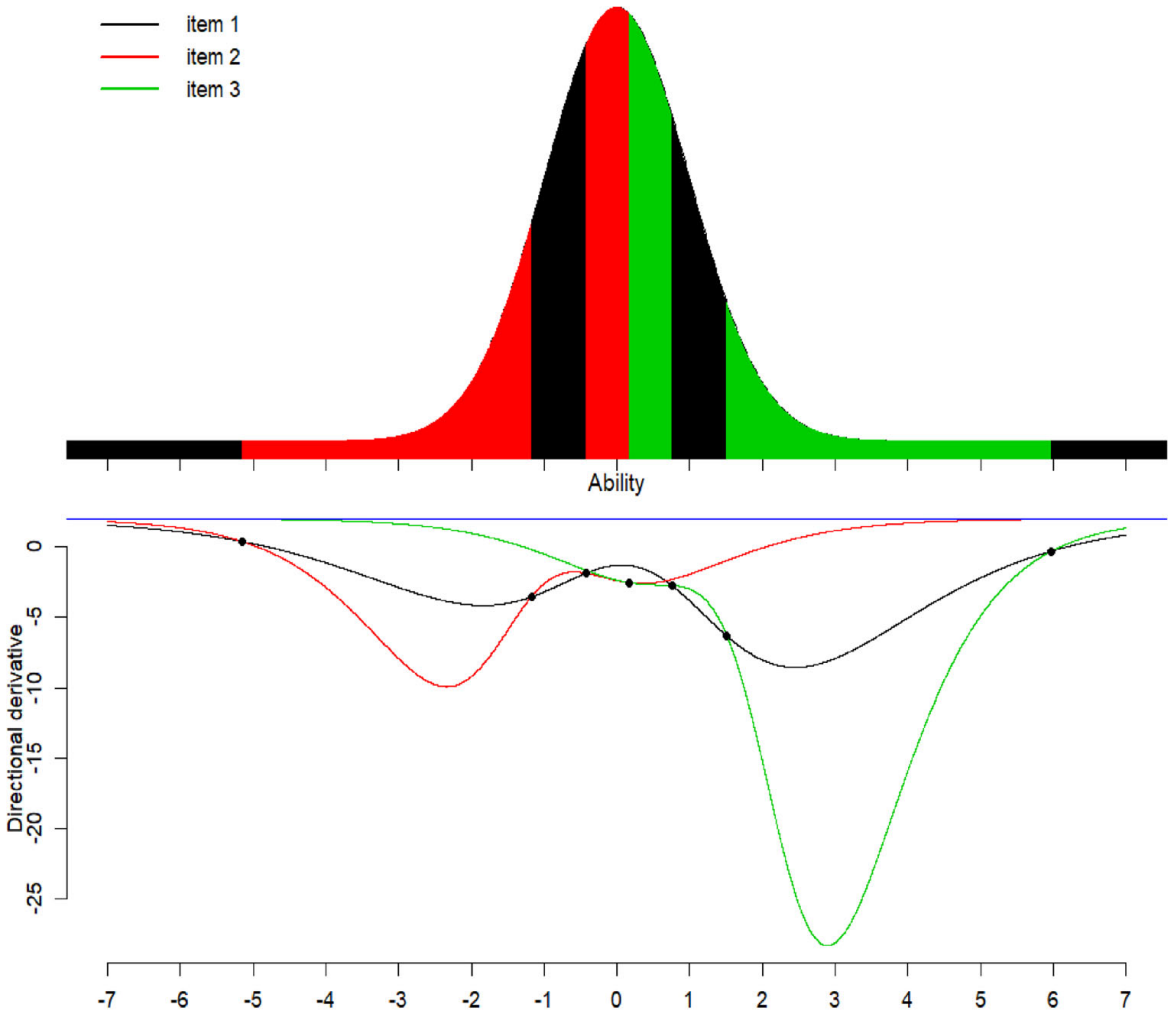
Calibration of Item 1, 2 and 3 by all examinees, see Fig. 7a.

Finally, we calibrate Item 1, 2 and 3 simultaneously using the population of examinees participating in the computerized test. The optimal unrestricted design recommends us to select approximately 16.67% of all available examinees at each of six optimal unrestricted design points of ability. For the optimal restricted design, we remarked in Sect. 2.3 that examinees with very high abilities should be assigned to the item with the lowest discrimination, here Item 1. For numerical computation, we assign therefore intervals $I_{10}=(-\infty ,\theta _{10U}]$ and $I_{1(K+1)}=[\theta _{1(K+1)L},\infty )$ to Item 1; between these intervals, we calculate an optimal *K*-interval design. It turns out that $K=2$ is sufficient here. The optimal restricted design suggests us to choose 11.97% and 23.17% of the total available examinees on the ability interval ($-5.147, -1.176$) and ($-0.424$, 0.170) for Item 2. Besides the intervals $(-\infty ,-5.147)$ and $(5.975,\infty )$ where almost no examine falls in, we choose 21.62% and 16.02% of examinees for Item 1 on the intervals ($-1.176, -0.424$) and (0.754, 1.513). Lastly, on the intervals (0.170, 0.754) and (1.513, 5.975) we select 20.70% and 6.82% of examinees for Item 3, see forth panel of Fig. 11. According to the Equivalence Theorem for Item Calibration the directional derivatives in the last panel of Fig. 11 show that the restricted design is optimal for selection of examinees based on their estimated abilities. The random design needs 32.04% more examinees to have the same efficiency as the restricted D-optimal design.

An alternative way of calibration would be the administration of all three items to all available examinees. The main “cost” of it is the increased testing time for each examinee which is three times larger (if we make the simplifying assumption that all items require the same testing time). The information from this design is three times the information of the random design and we can therefore use efficiencies with respect to the random design to compute efficiency of the all-examinees-all-items design versus the restricted D-optimal design. As written above, the restricted D-optimal design has 32.04% sample gain, or in other words, it has 1.3204 times the information of the random design. Therefore, the all-examinees-all-items design has $3/1.3204 = 2.27$ times the information of the restricted D-optimal design despite needing three times more time. Since one has in reality more than three items to calibrate (see Sect. 5), the time gain is usually important to achieve.

## Scaling Up the Method for Large Banks of New Items

An assumption we made was that each examinee can calibrate (at most) one item. We had examples with an item bank of three items. Realistic situations have often large banks of new items, and it is desired that each examinee calibrates several items. We will show now how one can easily use the methods described for realistic situations. We assume that the maximal number *k* of items which an examinee can calibrate is given by practical circumstances, e.g., the time necessary for the test. The number of new items *n* to calibrate is $>k$, such that we need to allocate them to different examinees. Let us assume for simplicity that *n* is a multiple of *k*. We divide the *n* items into *k* blocks of *n* / *k* items each. Each examinee is supposed to calibrate exactly one item per block. The blocking might be done taking content of items into account or simply randomly. We compute now for each of the *n* / *k*-item block the optimal restricted design separately. This gives us the optimal calibration with the additional restriction of this blocking.

We can compute the D-efficiency of the random design compared to the restricted optimal design for each block. It follows from formula (10) that the overall efficiency of the random design compared to the blocked restricted optimal design is the geometric mean of the block efficiencies.

## Discussion and Conclusion

Item calibration is an important tool for maintaining, updating and developing new items for an item bank. In the case of a two-parameter logistic model, the unrestricted D-optimal design for calibration of one new item has two optimal ability levels of examinees (${\theta } = b \pm \frac{{1.543}}{a}$) where one should sample equal proportions of the examinee population at these points. In practice, it is impossible to sample equal proportions of examinees from these optimal points of ability due to unavailability or limited availability of examinees. Sampling symmetrically around the optimal ability levels works in some situations. But in many cases, it is not clear how to define such symmetric designs, e.g., if optimal ability levels are too close to each other. To avoid possibly inefficient ad hoc solutions, we have used restricted optimal designs to calibrate new items where we used optimal intervals instead of points to sample the examinees from the population.

In this paper, we derived locally optimal designs. Their quality might depend on the quality of the prior guess about the item parameters. If the true item parameters are a little different from the prior guess, we have seen robustness; however, if the difference is large, the locally optimal design might be a bad choice. Therefore, alternatives to local optimality, Bayesian or maximin optimality, can be applied, see e.g., Atkinson et al. (2007), Chapter 17 and 18. Combination of these general optimal design approaches with the restricted optimality considered here could be an area of future research.

Further, an opportunity in computerized calibration is to re-estimate the item parameters from the ongoing calibration and to apply a sequential optimal design, see Lu (2014), van der Linden and Ren (2015) and Ren et al. (2017). This sequential and the Bayesian (or minimax) approach can also be combined. However, in tests, e.g., the Swedish Scholastic Assessment Test, all examinees are tested more or less simultaneously. If calibration items are added to tests where all examinees are tested more or less in parallel, we think therefore that a minimax or Bayesian approach should be used in a non-sequential context.

In this manuscript, we assume that abilities of examinees are well determined in the operational part of the test before it is decided which item to calibrate depending on their ability. We ignore here the fact that we use estimated abilities and not true abilities, but there is some uncertainty around the estimates: The examinee might be a bit better or worse than the estimated ability (the examinee might have had bad or good luck in the examination). However, the abilities should be reasonably well estimated if the operational part of the achievement test is large and calibration items are added toward the end of this test. Note that Ren et al. (2017) suggested to seed the new items into the final part of the test and He et al. (2019) concluded in their situation that even middle positions worked equally well. Nevertheless, for handling the uncertainty in abilities, it is conceptually possible to use the here described restricted optimal design approach in connection with using posterior distributions of abilities [see e.g., Section 2.1. of Ren et al. (2017)] rather than point estimates.

While our developed theory applies generally to item response models and to convex and differentiable optimality criteria, we have in the examples considered a two-parametric logistic model together with D-optimality. It might be interesting to explore the structure of optimal designs for other models. For example including a third parameter modeling a guessing probability has been advocated in this context of achievement tests, see e.g., van der Linden and Ren (2015). Further, the examinees’ abilities might not adequately be characterized by a one-dimensional ability parameter. Then a multidimensional IRT model might be considered. Optimal estimated designs for these models will be considered in future research where other optimality criteria will be considered as well.

Finally, we assumed in the Equivalence Theorem for Item Calibration that each examinee at most can calibrate one item. We described how this can be applied in a situation where everyone calibrates more items. This leads however to an optimal design under a blocking restriction. When there is no content-reason for a specific blocking and when the blocks are created randomly, it might be desirable to improve the design even more and to drop the blocking restriction. An extension of the Equivalence Theorem such that optimization can be done without the blocking restriction is a task for future research.

### Electronic supplementary material

Below is the link to the electronic supplementary material.

Supplementary material 1 (pdf 181 KB)

Supplementary material 2 (R 7 KB)

Supplementary material 3 (R 12 KB)

Supplementary material 4 (R 12 KB)

Supplementary material 5 (R 15 KB)

Supplementary material 6 (R 11 KB)

## Appendix: Proof of Theorem in Sect. 2.3

We have to prove: “$$h^*$$ is $$\Psi $$-optimal in $$\Xi ^g_s$$$$\Leftrightarrow $$ (7)”. Condition (7) means: $$P_{h^*} (\{(\theta ,i)\in {\tilde{\chi }}: F_\Psi (h^*,\theta ,i)>L(h^*,\theta ) \})=0 $$.

“$\Leftarrow$” Let ${\tilde{h}} \in \Xi ^g_s$ be arbitrary. We have

$$\begin{aligned} F_\Psi (h^*,{\tilde{h}}) = \sum _{i=1}^n F_\Psi (h^*,{\tilde{h}}_i) = \sum _{i=1}^n \int _{\Theta } F_\Psi (h^*,\theta ,i) {\tilde{h}}_i(\theta )~\mathrm{d}\theta . \end{aligned}$$

Since $F_\Psi (h,\theta ,i) \ge L(h,\theta )$, we have

$$\begin{aligned} F_\Psi (h^*,{\tilde{h}}) \ge \sum _{i=1}^n \int _\Theta L(h^*,\theta ) {\tilde{h}}_i(\theta )~\mathrm{d}\theta = \int _\Theta L(h^*,\theta ) \sum _{i=0}^n {\tilde{h}}_i(\theta )~\mathrm{d}\theta - \int _\Theta L(h^*,\theta ) {\tilde{h}}_0(\theta )~\mathrm{d}\theta . \end{aligned}$$

Since $$L \le c^*$$$$h^*$$-almost surely and the integral over $${\tilde{h}}_0$$ is $$1-s$$,

$$\begin{aligned} F_\Psi (h^*,{\tilde{h}})\ge & {} \int _\Theta L(h^*,\theta ) g(\theta )~\mathrm{d}\theta - c^* (1-s)= \int _\Theta L(h^*,\theta ) \sum _{i=1}^n h_i^*(\theta )~\mathrm{d}\theta \\&{\mathop {=}\limits ^{(7)}}&\sum _{i=1}^n \int _\Theta F_\Psi (h^*,\theta ,i) h^*_i(\theta )~\mathrm{d}\theta = \sum _{i=1}^n F_\Psi (h^*,h_i^*)= F_\Psi (h^*,h^*)=0 \end{aligned}$$

(the last equation follows directly from the definition of the directional derivative). This means that we have shown $F_\Psi (h^*,{\tilde{h}})\ge 0$ for any design ${\tilde{h}} \in \Xi ^g_s$ and the optimality of $h^*$ follows from a theorem of Whittle (1973), see also Theorem 3.6 of Silvey (1980) and Theorem A (i) of Sahm and Schwabe (2000).

“$$\Rightarrow $$” We will show this implication by contradiction. If (7) is not fulfilled, then there is some $$j \in \{0,1,\dots ,n\}$$ with $$P_{h_j^*} (\{\theta : F_\Psi (h^*,\theta ,j)>L(h^*,\theta ) \})>0$$ meaning that there are some $$j,k \in \{0,1,\dots ,n\}, j \ne k,$$ with $$ P_{h_j^*} (\{\theta : F_\Psi (h^*,\theta ,j)>F_\Psi (h^*,\theta ,k) \})>0.$$ Case 1: $$j,k \in \{1,\dots ,n\}$$. There has to be some set $$\Theta _0 \subseteq \Theta $$ with $$F(h^*,\theta ,j)>F(h^*,\theta ,k)$$ for all $$\theta \in \Theta _0$$ and $$P_{h_j^*} (\Theta _0)>0$$, i.e., item *j* is sampled on $$\Theta _0$$ despite there is another item, *k*, which has a smaller directional derivative. We construct a new design $$h^{**}$$ by moving all sample mass on $$\Theta _0$$ from item *j* to item *k*:

$$\begin{aligned} h^{**}(\theta ,i)= & {} h^*(\theta ,i) \hbox { if } \theta \in \Theta \backslash \Theta _0 \hbox { or } i \not \in \{j,k\}, \\ h^{**}(\theta ,k)= & {} h^*(\theta ,k)+h^*(\theta ,j) \hbox { and } h^{**}(\theta ,j) = 0 \hbox { if } \theta \in \Theta _0 . \end{aligned}$$

It is clear that $$ h^{**} \in \Xi ^g_s$$. We have then

$$\begin{aligned} F_\Psi (h^*,h^{**}) = F_\Psi (h^*,h^*) + \int _{\Theta _0} \left\{ F_\Psi (h^*,\theta ,k) - F_\Psi (h^*,\theta ,j) \right\} h^{**}(\theta ,j)~\mathrm{d}\theta < 0 \end{aligned}$$

where the inequality holds since $$F(h^*,h^*)=0$$ and since the integral is negative due to the fact that $$F_\Psi (h^*,\theta ,k) - F_\Psi (h^*,\theta ,j)$$ is negative. Since we have found an $$h^{**} \in \Xi ^g_s$$ with $$F_\Psi (h^*,h^{**})<0$$, it follows that the design $$h^*$$ cannot be $$\Psi $$-optimal in $$\Xi ^g_s$$, according to the theorem of Whittle (1973).

Case 2: $$j=0, k \in \{1,\dots ,n\}$$. There has to be some set $$\Theta _0 \subseteq \Theta $$ with $$c^*>F_\Psi (h^*,\theta ,k)$$ for all $$\theta \in \Theta _0$$ and $$P_{h_0^*} (\Theta _0)>0$$, i.e., there is some positive non-sample probability where the directional derivative of item *k* is below $$c^*$$. From the definition of $$c^*$$ follows that if one samples everywhere where $${\tilde{L}} < c^*$$, then the sampling mass is $$\le s$$. Therefore, if no full sampling is done on $$\Theta _0 \subseteq \{\theta : L < c^*\}$$, there has to be sampling on $$\{\theta : L=c^*\}$$. Formally, this means that we can find $$\Theta _1 \subseteq \{\theta : L=c^*\}$$ such that $$ \int _{\Theta _0} h_0(\theta )~\mathrm{d}\theta = \int _{\Theta _1} (g(\theta )-h_0(\theta ))~\mathrm{d}\theta .$$ We can then construct a competing design $$\xi ^{**}$$ by moving sampling mass from $$\Theta _1$$ to item *k* on $$\Theta _0$$:

$$\begin{aligned} h^{**}(\theta ,0)= & {} \sum _{i=0}^n h^*(\theta ,i) \hbox { and } h^{**}(\theta ,1)=\dots =h^{**}(\theta ,n)=0 \hbox { if } \theta \in \Theta _1, \\ h^{**}(\theta ,k)= & {} h^*(\theta ,k)+h^*(\theta ,0) \hbox { and } h^{**}(\theta ,0) = 0 \hbox { if } \theta \in \Theta _0,\\ h^{**}= & {} h^*, \hbox { otherwise}. \end{aligned}$$

One can show $$h^{**} \in \Xi ^g_s; F_\Psi (h^*,h^{**})<0$$. Non-optimality of $$h^*$$ follows like in Case 1.

Case 3: $$j \in \{1,\dots ,n\}, k=0$$. A competing design $$h^{**}$$ with $$F_\Psi (h^*,h^{**})<0$$ can be constructed similarly like in Case 2.
