# Supplementary material for: Optimal Item Calibration for Computerized Achievement Tests
Source: Psychometrika. 2019 Jun 10;84(4):1101–28. doi: 10.1007/s11336-019-09673-6 (PMC6820328; doi:10.1007/s11336-019-09673-6)
Supplement: Supplementary file 1 — Supplementary material 1 (pdf 181 KB) [file 11336_2019_9673_MOESM1_ESM.pdf]

Supplementary Material for Section 3

Robustness of the locally D-optimal restricted designs

The locally D-optimal restricted design for Item 1 (assumed $a = 1, b = 0.5$) and $s = 0.1$ is as noted in the main paper:

$$h_1(\theta) = \begin{cases} g(\theta), & \text{if } -1.215 \leq \theta \leq -0.984 \text{ or } 1.600 \leq \theta \leq 2.577, \\ 0, & \text{otherwise.} \end{cases} \quad (1)$$

We calculate the relative D-efficiency of this design versus the random design (which allocates examinees to the item with probability s). It is 1.3338.

Now, assuming that the true parameter values are different from the planning values, we calculate again the relative efficiency of design (1) versus the random design. Figure 1 shows for true discrimination $a \in [0.1, 2]$ and true difficulty $b \in [-1, 2]$ the relative efficiency as contour plot. We see that the locally D-optimal design is better than the random design (relative efficiency>1) for many cases of true a and b. Even if parameters are slightly misspecified, one has still a design which is better than the random design.

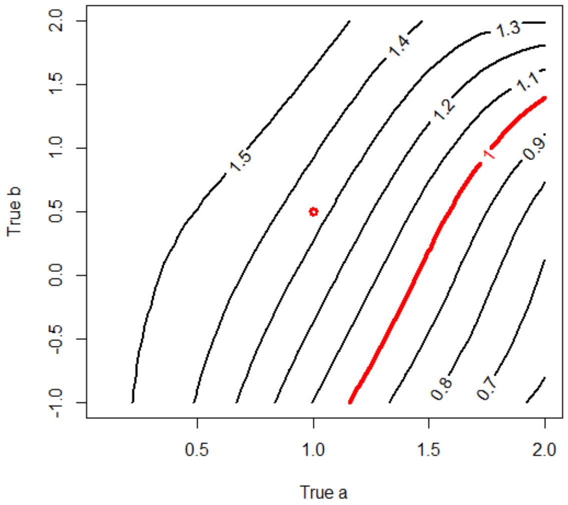

Figure 1: Robustness of locally D-optimal restricted ($s = 0.10$) design for calibration of Item 1: Contour plot of relative efficiency versus random design; efficiencies > 1 indicate better performance than random design; red dot: situation when assumed and true parameter values equal

The locally D-optimal restricted design for Item 2 (assumed $a = 1.5, b = -1.2$) and $s = 0.25$ is as noted in the main paper:

$$h_1(\theta) = \begin{cases} g(\theta), & \text{if } -3.592 \le \theta \le -1.200 \text{ or } -0.061 \le \theta \le 0.282, \\ 0, & \text{otherwise.} \end{cases} \quad (2)$$

For true $a \in [0.5, 2.5]$ and $b \in [-3, 0.5]$, the contour plot for relative efficiency of design (2) versus the random design is shown in Figure 2. The computed design is still better than the random design, if the parameters are misspecified but the true b is approximately smaller than -0.4.

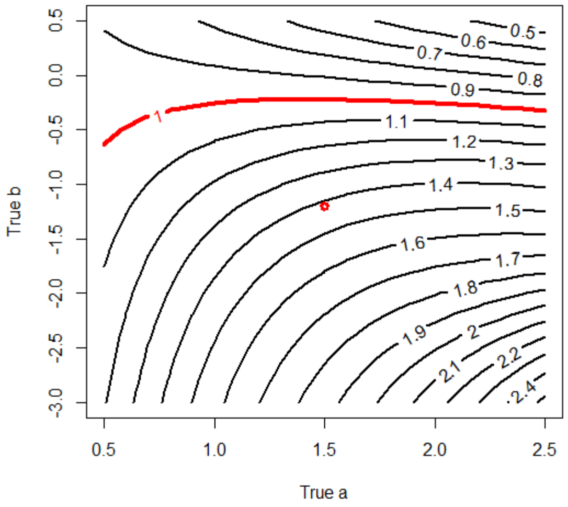

Figure 2: Robustness of locally D-optimal restricted ($s = 0.25$) design for calibration of Item 2: Contour plot of relative efficiency versus random design; efficiencies > 1 indicate better performance than random design; red dot: situation when assumed and true parameter values equal

The locally D-optimal restricted design for Item 3 (assumed $a = 1.6, b = 2$) and $s = 0.35$ is as noted in the main paper:

$$h_1(\theta) = \begin{cases} g(\theta), & \text{if } 0.043 \leq \theta \leq 0.611 \text{ or } 1.091 \leq \theta \leq 5.417, \\ 0, & \text{otherwise.} \end{cases} \tag{3}$$

For true $a \in [0.5, 2.5]$ and $b \in [0.5, 3.5]$, the contour plot for relative efficiency of design (3) versus the random design is shown in Figure 3. The computed design is still better than the random design, e.g. if a is correct and b is misspecified but larger than ≈ 0.6, or if b is correct and a larger than ≈ 0.75.

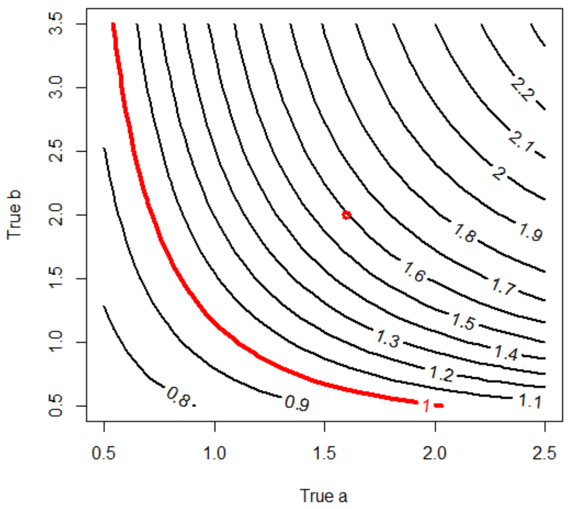

Figure 3: Robustness of locally D-optimal restricted ($s = 0.35$) design for calibration of Item 3: Contour plot of relative efficiency versus random design; efficiencies > 1 indicate better performance than random design; red dot: situation when assumed and true parameter values equal

We conclude from these scenarios that as long as the parameters are not severely misspecified, the locally D-optimal restricted design is still better than a random design.

Cases when one unrestricted design point falling outside the interval of the restricted design

We present here two figures using all combinations of discrimination parameter $a \in \{0.3, 0.4, \ldots, 2\}$ and difficulty parameter $b \in \{0, 0.1, \ldots, 2\}$ for construction of locally D-optimal restricted design to calibrate one item for sample proportion $s = 0.20$ and $s = 0.35$. The solid points indicate combinations of a and b for which the lower interval of the restricted design does not contain the left point of the unrestricted design. If we consider all combinations of $a \in \{0.3, 0.4, \ldots, 2\}$ with negative difficulty parameter $b \in \{0, -0.1, \ldots, -2\}$ for sample proportion $s = 0.20$ and $s = 0.35$, we will get the mirror images of these figures and in these cases the solid dots represent combinations of a and b for which the upper interval of the restricted design does not contain the right value of the unrestricted design.

One may conclude with these figures that if the item has difficulty around the center ($b = 0$) of ability distribution, the unrestricted D-optimal design contains the unrestricted design points. As the value of the difficulty parameter moves away from $b = 0$ for a fixed discrimination parameter a, we encounter the situation where the restricted optimal design interval does not contain one of the unrestricted design points.

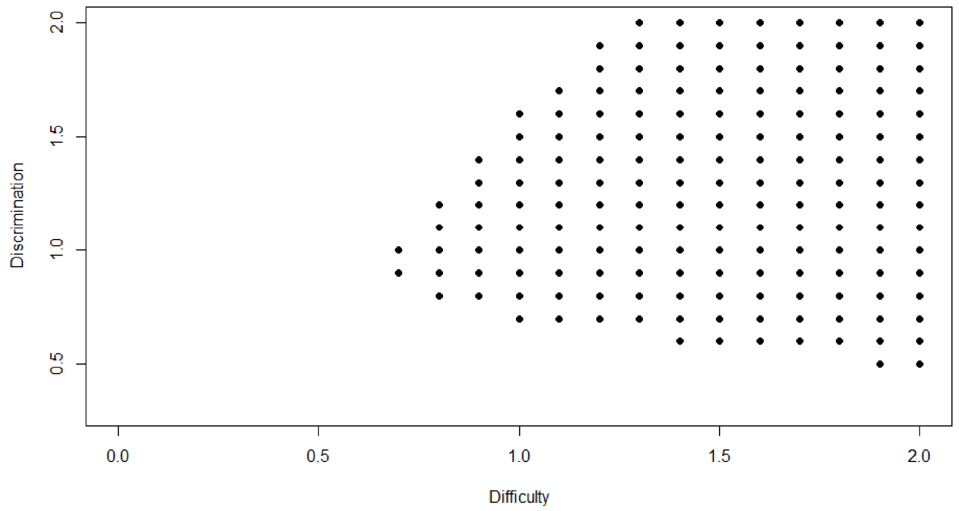

Figure 4: For sample proportion $s = 0.20$. The solid points indicate values of a and b for which the lower interval of the restricted design does not contain the lower value of the unrestricted design.

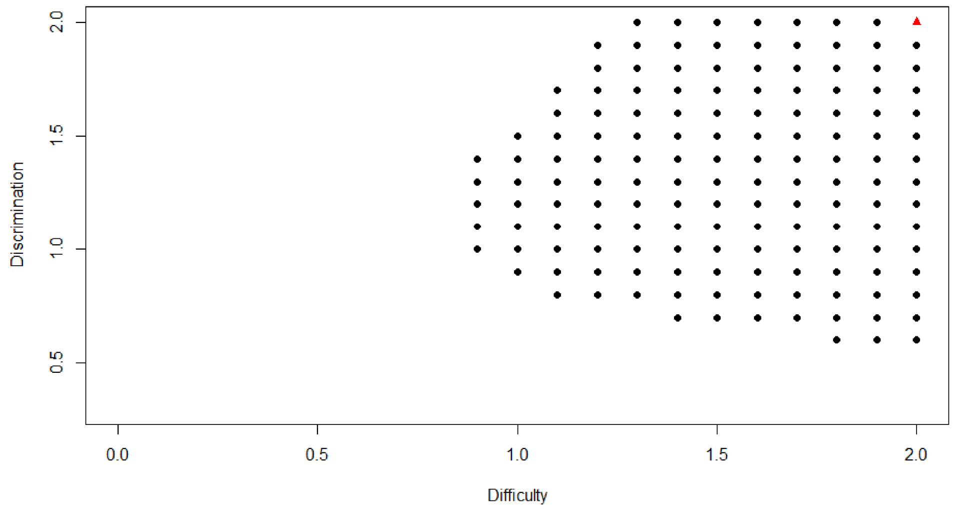

Figure 5: For sample proportion $s = 0.35$. The solid points indicate values of a and b for which the lower interval of the restricted design does not contain the lower value of the unrestricted design. The red triangle depicts that the restricted design is a one interval solution.
